# Optical characterization of stratified-premixed natural gas direct-injection combustion regimes

**DOI:** 10.1177/14680874221107188

**Published:** 2022-07-02

**Authors:** Jeremy Rochussen, Matthew Knight, Gibson Clark, Patrick Kirchen

**Affiliations:** Department of Mechanical Engineering, The University of British Columbia, Vancouver, BC, Canada

**Keywords:** Direct injection, natural gas, optical, stratified, injection timing, pilot ignited, fuel mixing, chemiluminescence, partially premixed

## Abstract

Gaseous fuels for heavy-duty internal combustion engines provide inherent advantages for reducing CO_2_, particulate matter (PM), and NO_X_ emissions. Pilot-ignited direct-injected NG (PIDING) combustion uses a small pilot injection of diesel to ignite a late-cycle main direct injection of NG, resulting in significant reduction of unburned CH_4_ emissions relative to port-injected NG. Previous works have identified NG premixing as a critical parameter establishing indicated efficiency and emissions performance. To this end, a recent experimental investigation using a metal engine identified six general regimes of PIDING heat release and emissions behavior arising from variation of NG stratification through control of relative injection timing (RIT) of the NG with respect to the pilot diesel. The objective of the current work is to provide comprehensive description of in-cylinder fuel mixing of direct injected gaseous fuel and its impacts on combustion and pollutant formation processes for stratified PIDING combustion. In-cylinder imaging of OH*-chemiluminescence (OH*-CL) and PM (700 nm), and measurement of local concentration of fuel is considered for 11 different RIT , representing 5 regimes of stratified PIDING combustion (performed with $P_{\mathrm{inj}}=22.0$ MPa and ϕ=0.63 ). The magnitude and cyclic variability of premixed fuel concentration near the bowl wall provides direct experimental validation of thermodynamic metrics ( $\mathrm{RI}T_{\mathrm{premix}}$ , $\mathrm{SO}I_{\mathrm{NG},\mathrm{trans}}$ , $\mathrm{RI}T^{*}$ ) that describe the fuel-air mixture state of all 5 regimes of PIDING combustion. The local fuel concentration develops non-monotonically and is a function of RIT. High indicated efficiency and low CH_4_ emissions previously observed for stratified-premixed PIDING combustion in previous (non-optical) investigations are due to: (i) very rapid reaction zone growth ( >45 m/s) and (ii) more distributed early reaction zones when overlapping pilot and NG injections cause partial pilot quenching. These results connect and extend the findings of previous investigations and guide the future strategic implementation of NG stratification for improved combustion and emissions performance.

## Introduction

On-road freight activity is forecasted to grow 25% by 2030 and is estimated to already account for 7% of world energy-related CO_2_ emissions.^1,2^ The stringent energy density requirements for long-haul trucking represents a major challenge to the wide-spread electrification in this sector and motivates the development of advanced propulsion technologies for the short- and medium-term which reduce greenhouse gas (GHG) emissions.^3^

Life-cycle analysis indicates a net reduction of GHG emissions of 10%–15% is realistic for heavy-duty vehicles where diesel is replaced by NG in addition to reduction of particulate matter (PM) and NO_X_ emissions.^4–6^ Development of NG propulsion technologies also represents a commercially attractive pathway for application of more deeply decarbonized gaseous fuels such as renewable NG (RNG) and hydrogen, which are still immature in terms of infrastructure and technical readiness.

Because the main constituent of NG, CH_4_, is a potent GHG,^4^ emissions of unburned hydrocarbons (uHCs) from NG engines is an important challenge that must be addressed.^7,8^ Several premixed (i.e. port-injected) NG combustion concepts such as reactivity-controlled compression ignition and split diesel injections have been shown as valuable approaches for reducing uHC emissions and increasing efficiency with limited penalty to NO_X_ at both low- and high-load conditions.^9,10^ Pilot-ignited direct-injection NG (PIDING) combustion is another concept, which uses a late-cycle pilot injection of diesel (approximately 5% of total fuel energy) followed by a main injection of NG. Typically, the main PIDING combustion process is non-premixed, which allows for higher compression ratios, providing high efficiency and very low uHC emissions at the cost of increased PM and NO_X_ emissions relative to highly premixed (i.e. port-injected) NG systems.^5,11^

In PIDING combustion, a portion of the NG also reacts in a rapid partially-premixed mode in parallel to establishment of a quasi-steady jet flame.^12^ The fraction of fuel converted in the partially-premixed fraction, $f_{\mathrm{premix}}$ , is predominantly defined by the time available for premixing prior to ignition, which can be manipulated through changes in the relative injection timing ( RIT ) of the NG with respect to the pilot, defined as:

(1) $$\mathrm{RIT}=\mathrm{SO}I_{\mathrm{NG}}-\mathrm{SO}I_{\mathrm{pilot}}$$

where $\mathrm{SO}I_{\mathrm{NG}}$ and $\mathrm{SO}I_{\mathrm{pilot}}$ are the commanded start of NG and diesel pilot injection, respectively. Several investigations have demonstrated that significant advantages in emissions and efficiency can be achieved by intentionally increasing the NG premixing time and promoting more premixed combustion by reducing the RIT to negative values (i.e. NG injection prior to pilot injection). To this end, two general approaches have been considered: (i) slightly premixed combustion (SPC) modes using a small reduction of RIT from conventional non-premixed PIDING values (e.g. Faghani et al.^13^ and McTaggart-Cowan et al.^14,15^) and (ii) stratified-premixed PIDING modes where one or more NG injections are performed during the compression stroke to generate highly premixed conditions (e.g. Florea et al.^16^, Neely et al.,^17^ Li et al.,^18^ and Munshi et al.^19^). For both these approaches, late-cycle $\mathrm{SO}I_{\mathrm{pilot}}$ is used for fast-response combustion phasing control.

SPC was studied with −2 ms < RIT <+2 ms (operating conditions with RIT<0 were designated SPC modes) and an optimized SPC mode achieved a 90% reduction in PM mass and a 2% increase of gross indicated efficiency, $\eta_{i,g}$ , relative to a conventional high-load PIDING operating condition. This optimized SPC mode had negligible penalty to NO_X_ or CH_4_ emissions, however with advancing $\mathrm{SO}I_{\mathrm{NG}}$ emissions of both NO_X_ and CH_4_ increased significantly.^13^ The decreased PM emissions were attributed to the increased NG premixing time prior to NG ignition resulting in less NG with an equivalence ratio, ϕ , in the PM formation region (2 <ϕ< 5). In earlier studies, increased efficiency and reduction of CO and PM was also observed for similar SPC operating conditions ( RIT>−3° ), which were attributed to higher peak apparent heat release rates (AHRR) and reduced combustion duration.^14,15^ The major drawbacks of the SPC mode were indicated to be increased cycle-to-cycle variability (CCV, measured as COV of peak cylinder pressure, $P_{\mathrm{cyl}}$ ) and combustion harshness (maximum rate of pressure rise, RoPR), and moderate increases of CH_4_ emissions at medium and low load.^14,15^

Stratified-premixed PIDING combustion was investigated by advancing $\mathrm{SO}I_{\mathrm{NG}}$ up to −34 CAD after top dead center (aTDC) such that the end of NG injection ( $\mathrm{EO}I_{\mathrm{NG}}$ ) occurs well before pilot injection and auto-ignition. This strategy has been termed co-direct injection (DI^2^) and demonstrated increased efficiency with decreased PM and CO relative to non-premixed PIDING combustion and acceptable CCV (COV of indicated mean effective pressure limited to less than 2%).^16^ Based on numerical simulation, the authors describe the $DI^{2}$ combustion as rapid flame propagation through a stratified NG-air mixture, which initiates at the pilot ignition regions. The flame propagation occurs in parallel to NG-air mixing processes driven by both diffusion and jet-induced turbulence.^16^ Relative to port-injected NG operation, a 75% reduction in the emissions of CH_4_ was achieved. However, CH_4_ emissions rapidly increased for $\mathrm{SO}I_{\mathrm{NG}}<-34$ CAD aTDC due to impingement of the NG fuel jets outside of the piston bowl.^16,17^ Emissions of NO_X_ were also observed to be significantly higher for $DI^{2}$ relative to comparable non-premixed PIDING combustion. In light of the very low PM emissions of $DI^{2}$ , EGR was considered a viable method to reduce NO_X_, in agreement with earlier experimental investigations.^14,16^

Splitting the main NG injection into early- and late-cycle injections has also been considered as a strategy to control NG stratification.^18,19^ Increased efficiency and a reduction of PM and CO were reported for these strategies, although limiting uHC emissions was a challenge. These investigations indicated that the increased turbulent mixing rates produced by the late NG injection supported higher flame propagation speeds and reduced CH_4_ emissions from slow flame extinction,^19^ which has also been noted for DISI combustion.^20–22^

Investigations of stratified-PIDING combustion have used $\mathrm{SO}I_{\mathrm{NG}}$ to control the NG residence time prior to combustion and therefore the degree of NG stratification. This has been quantified using either RIT ,^12–15^ and/or the time delay between NG injection and the start of partially-premixed NG combustion, $\theta_{\mathrm{SOC},\mathrm{NG}}$ .^12,23,24^ In-cylinder OH*-chemiluminescence (OH*-CL) imaging of PIDING combustion with −6 ms <RIT< +1.5 ms indicated that the leading edge of the main AHRR peak coincided with start of partially-premixed NG combustion and that this is an appropriate metric for $\theta_{\mathrm{SOC},\mathrm{NG}}$ .^12^ A subsequent non-optical measurement campaign defined $\theta_{\mathrm{SOC},\mathrm{NG}}$ as the phasing at which the AHRR reaches 20% of its maximum value for −26.5 ms <RIT<+3.0 ms:

(2) $$\theta_{\mathrm{SOC},\mathrm{NG}}=\theta {|}_{\mathrm{AHRR}=0.2\cdot \max(\mathrm{AHRR})}$$

where $\theta_{\mathrm{SOC},\mathrm{NG}}$ was used to define the NG premixing time, $\tau_{\mathrm{NG}}$ :

(3) $$\tau_{\mathrm{NG}}[\mathrm{ms}]=\theta_{\mathrm{SOC},\mathrm{NG}}-\mathrm{SO}I_{\mathrm{NG}}$$

**Figure 1.**
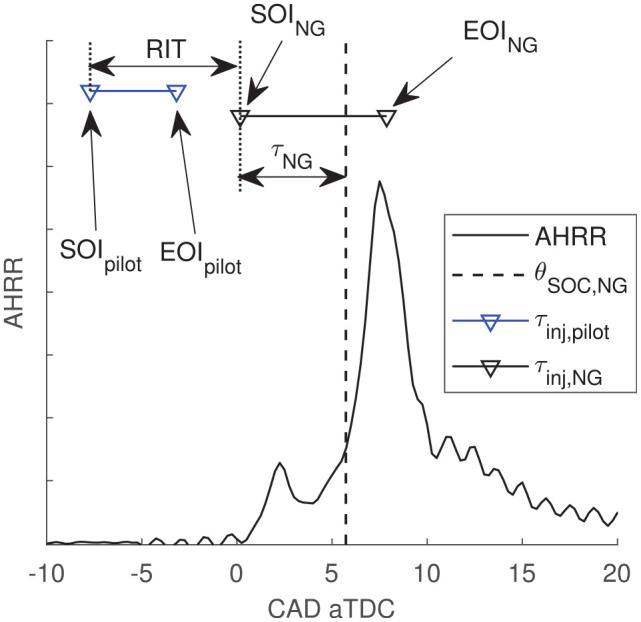
Definitions for key PIDING injection and combustion phasings.

These previously introduced metrics to characterize the NG premixing are graphically summarized in Figure 1 with a sample measurement of AHRR for a typical (non-premixed) PIDING operating condition ( RIT=+1 ms).

All investigations of stratified PIDING combustion have noted transitions in the characteristic behavior of PIDING combustion for different degrees of NG premixing (e.g. transition of PM sensitivity to RIT for non-premixed PIDING vs SPC^14,25^). However, the majority of published investigations are limited to subsets of the full spectrum of NG premixing that is possible with PIDING fuel systems, where RIT is continuously variable. To connect the findings of these investigations and develop a framework of generally-relevant (i.e. not engine-specific) PIDING combustion regimes, a systematic evaluation of stratified-PIDING combustion and emissions spanning from fully-premixed NG to non-premixed PIDING combustion was recently conducted.^24^ The identified regimes of PIDING combustion motivate and guide the current work and are summarized below.

### Regimes of stratified PIDING combustion

To classify regimes of PIDING combustion, AHRR and emissions behavior was analyzed for −26.5 ms ≤RIT≤ +3.0 ms with ϕ=0.47−0.71 , and $P_{\mathrm{inj}}=14-22$ MPa in a previous study employing an all-metal engine.^24^ A constant engine speed (1000 RPM) was considered, so the range of RIT is equivalently expressed on a crank angle basis as −159°≤RIT≤+18° .

**Figure 2.**
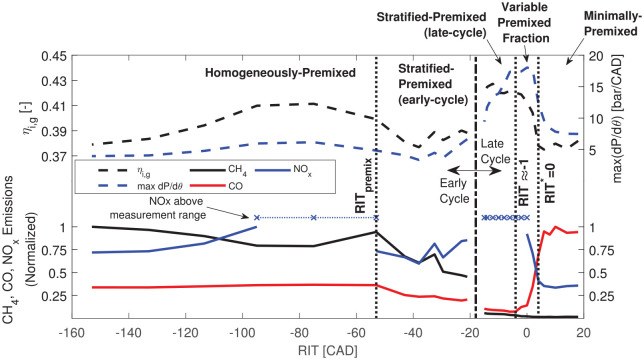
Summary of PIDING emissions and engine performance metrics across spectrum of NG premixing ( ϕ=0.63 , $P_{\mathrm{inj}}=22$ MPa).

Experimental results of combustion and emissions performance across the full range of NG premixing conditions considered in the all-metal investigation are presented in Figure 2.^24,26^ Combustion regimes were considered to be domains of RIT (representing NG stratification) where relevant heat release features (e.g. combustion duration, ignition delay, efficiency) and emissions responded in the same manner to major engine control parameters ( $P_{\mathrm{inj}}$ , RIT , ϕ ). Combustion regime domains for the operating condition shown in Figure 2 ( ϕ=0.63 , $P_{\mathrm{inj}}=22$ MPa) are also identified with dotted vertical lines. Combustion and emissions behavior of the minimally-premixed, variable-premixed fraction, and stratified-premixed (late-cycle) regimes align with results in the literature of non-premixed PIDING,^5,11^ SPC,^13–15^ $DI^{2}$ ,^16,17,27^ and port-injected dual-fuel^28^ combustion strategies, respectively. Of particular interest is the stratified-premixed (late-cycle) regime (termed $DI^{2}$ elsewhere), where low CO and CH_4_ emissions are achieved with high efficiency and moderate combustion harshness.

**Figure 3.**
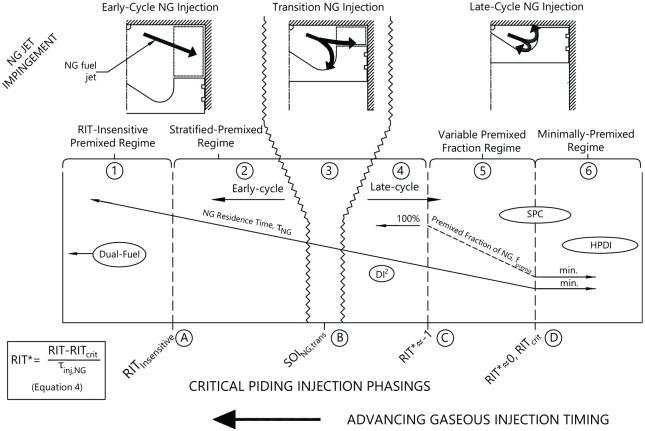
Summary of identified regimes of AHRR and emissions behavior of stratified-PIDING combustion with respect to critical injection phasings for −153°<RIT<+18° , 0.47<ϕ<0.71 , and $14\leq P_{\mathrm{inj}}\leq 22$ MPa. Figure adapted from Rochussen et al.^24^

In Figure 3, a generalized summary of the 6 identified regimes of PIDING combustion (①→⑥) and 4 critical injection phasings distinguishing transitions between the regimes (Ⓐ→Ⓓ) is presented. These injection phasings were determined such that they are general to a wide range of operating conditions ( ϕ and $P_{\mathrm{inj}}$ ) and are intended to provide a common framework within which to compare and investigate the effects of NG stratification on direct-injected NG combustion performance.

All combustion regimes were classified as either early- or late-cycle regimes based on whether NG jet impingement occurs inside (late-cycle) or outside the piston bowl (early-cycle), which significantly influenced injection control strategy, combustion behavior, and emissions as noted in other investigations.^16,17^ Near the transition between early- and late-cycle NG injection ( $\mathrm{SO}I_{\mathrm{NG}}\approx \mathrm{SO}I_{\mathrm{NG},\mathrm{trans}}$ Ⓑ) poor engine performance (combustion stability and emissions) occurs.^16,24^

Two early-cycle PIDING regimes were distinguished by $\mathrm{RI}T_{\mathrm{insens}.}$ (Ⓐ, $\mathrm{RI}T_{\mathrm{insens}.}\approx -53{}^{\circ}$ ): (i) The “RIT-Insensitive Premixed Regime” (①) for $\mathrm{RIT}<\mathrm{RI}T_{\mathrm{insens}.}$ , emissions were not significantly affected by changes in RIT and combustion and emissions behavior was observed to be consistent with port-injected dual-fuel combustion, and (ii) the “Stratified-Premixed Regime” (early-cycle) (②) for $\mathrm{RIT}>\mathrm{RI}T_{\mathrm{insens}.}$ , where RIT had a significant influence on combustion and emissions.

For late-cycle operating conditions ( $\mathrm{SO}I_{\mathrm{NG}}>\mathrm{SO}I_{\mathrm{NG},\mathrm{trans}}$ ), ignition, main combustion, and emissions behavior were very sensitive to $P_{\mathrm{inj}}$ and RIT . Defining general late-cycle PIDING combustion regimes valid for $P_{\mathrm{inj}}=14-22$ MPa and ϕ=0.47−0.71 required RIT to be scaled by the NG injection duration:

(4) $$\mathrm{RI}T^{*}=\frac{\mathrm{RIT}-\mathrm{RI}T_{\mathrm{crit}}}{\tau_{\mathrm{inj},\mathrm{NG}}}$$

where $\tau_{\mathrm{inj},\mathrm{NG}}$ is the NG injection duration (see Figure *fig : PIDING_Definitions_*, ${\mathrm{RIT}}^{*}$ is the scaled RIT is the minimum RIT remains at the minimum value observed for typical non-premixed PIDING and is not sensitive to RIT . In a previous investigation, ${\mathrm{RIT}}_{\mathrm{crit}}=0.68\pm 0.17$ was measured for all operating conditions covered in the current work. *rochussen* 2021 However, it should be noted that this value of ${\mathrm{RIT}}_{\mathrm{crit}}$ is expected to be sensitive to injector geometry and engine speed, and is therefore application specific.

The three late-cycle combustion regimes were identified using $\mathrm{RI}T^{*}$ : (i) The “Stratified-Premixed Regime” (late-cycle) (④) is characterized by $\mathrm{RI}T^{*}<-1$ (Ⓒ) where the NG injection is sufficiently early that $\mathrm{EO}I_{\mathrm{NG}}$ occurs well before the start of combustion ( $\theta_{\mathrm{SOC},\mathrm{NG}}$ ) and combustion and emissions behavior is consistent with DI^2^ combustion.^16,17^ For $-1\sim RIT^{*}<0$ , overlapping pilot and NG injections and $\tau_{\mathrm{NG}}=f(\mathrm{RIT})$ indicated that $f_{\mathrm{premix}}=f(\mathrm{RIT})$ . This regime was labeled the “Variable Premixed Fraction Regime” (⑤) and combustion and emissions behavior was consistent with SPC behavior.^13,14^ For a large portion of the variable premixed fraction regime, the overlapping pilot and NG injections resulted in quenching of the pilot by the NG jets. For $\mathrm{RI}T^{*}>0$ (Ⓓ), $\tau_{\mathrm{NG}}$ was at a minimum value and insensitive to RIT . This range of RIT includes typical non-premixed PIDING applications and is labeled the “Minimally-Premixed Regime” (⑥).

The above description of the spectrum of stratified PIDING combustion connects investigations of different stratified PIDING combustion strategies into a single generalized framework. To develop and refine this framework into a useful conceptual tool for PIDING, complementary measurements investigating the stratification and structure of NG mixing and combustion processes are needed. To address this gap, investigators have performed in-cylinder imaging of PIDING combustion processes following one of two general approaches: (i) optically-accessible engines fitted with production multi-jet PIDING injectors,^12,29–32^ or (ii) more fundamental investigations of single pilot-NG jet pairs in rapid compression/expansion machines (RCEMs).^23,33,34^ These investigations have been valuable for characterizing the structure of typical non-premixed PIDING combustion, however only a subset of these investigations address PIDING combustion with $\mathrm{RI}T^{*}<0$ .

Study of single pilot-NG jet pairs in RCEMs has demonstrated that RIT and the geometric injection angle between the pilot and NG jets has significant impact on both pilot and NG ignition.^23,33,34^ In particular, quenching of the pilot reactants by the cold NG jet has shown to increase variability in the ignition phasing and location of both fuels, which impacts NG premixing and main combustion behavior, as has been noted in optical engine experiments.^12,29,31^ Crucially, these fundamental studies only consider unbounded NG jets, and do not provide insight to the effects of NG jet impingement on combustion chamber surfaces (i.e. the piston bowl).

Decreasing RIT from the minimally-premixed regime such that the pilot injection is timed to ignite the tail of the NG jet (i.e. negative RIT ) was demonstrated to entrain the diesel pilot by the NG jet in an optical engine.^30^ This resulted in increased NG premixing and more rapid heat release. In-cylinder OH*-CL imaging for a slightly wider range of RIT has demonstrated that the main combustion process changes from a quasi-steady jet flame, to rapid distributed-ignition, to flame propagation as the RIT is adjusted between +1.3, +0.3, and −1.0 ms, respectively. For the same measurement conditions, pyrometric imaging indicated significant reduction of in-cylinder soot production as RIT was decreased,^32^ which corroborates numerical modeling results.^13^

RCEM and optically-accessible engine measurements of stratified PIDING combustion to date have provided significant insight to the role of pilot-NG interactions and the effects of increased NG premixing on PIDING combustion behavior. However, the range of NG premixing conditions investigated is narrowly focused on the transition between the minimally-premixed and variable premixed fraction regimes. Additional consideration of the role of combustion chamber geometry is needed as this is a critical parameter for stratified PIDING combustion.^16,17,24^ Finally, comparison of in-cylinder NG stratification and reaction zone structures to combustion and emissions performance is needed.

### Objectives and outline

The objectives of this work are to: (i) support and refine the previously identified regimes of PIDING combustion and associated critical injection phasings and (ii) describe the in-cylinder mixing processes of direct injected gaseous fuel and its impacts on combustion and in-cylinder pollutant formation processes. These objectives are addressed by applying in-cylinder imaging and local fuel concentration measurements to stratified PIDING combustion conditions ranging from homogeneously-premixed to nominally non-premixed in an optically-accessible engine.

The optical research engine facility, measurement diagnostics, and selected stratified PIDING combustion conditions are described first. Discussion of results is divided into 3 parts addressing adjacent domains of the spectrum of stratified PIDING combustion: (i) Minimally-premixed and variable premixed fraction regimes, (ii) Variable premixed fraction and stratified-premixed (late-cycle) regimes, and (iii) Early-cycle regimes. Last, a summary of the important in-cylinder processes is presented for all combustion regimes.

## Experimental facility and methods

**Figure 4.**
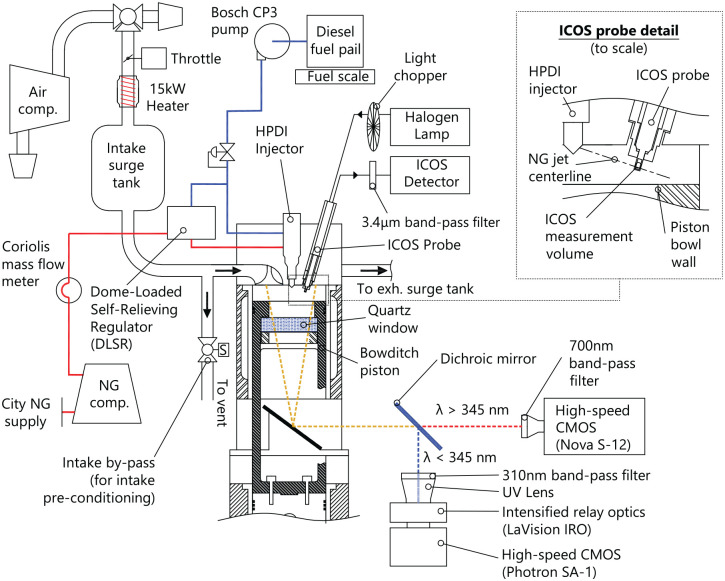
Single-cylinder optical engine facility. LaVision ICOS and high-speed imaging systems configuration shown. Note that ICOS measurement volume is located between two adjacent NG injection axes (see Figure 5).

The experimental facility used in this investigation is based on a 2.0 L, single-cylinder, optically-accessible Ricardo Proteus engine, the specifications of which are given in Table 1. This facility is operated in either an optically-accessible configuration with a Bowditch piston and quartz window, or in a conventional all-metal configuration (thermodynamic configuration). The current investigation only considers the optical engine configuration, however recently published measurements collected using the thermodynamic engine configuration were used to guide the current work and provide complementary measurement of fuel, air, and exhaust emissions flowrates.^24^ Details of the optical engine configuration are presented in Figure 4.

**Table 1.** Engine specifications and engine set-points common to all measurement conditions. Operating conditions for the thermodynamic configuration correspond to previously published measurements.^24^

Parameter	Thermodynamic	Optical
Displacement [L]	2.0	2.0
Bore [mm]	130	130
Stroke [mm]	150	150
Compression ratio [-]	13.25:1	12.6:1
Piston bowl shape	Eccentric torroid	Eccentric cylinder
Speed [rpm]	1000	1000
$T_{\mathrm{intake}}$ [°C]	40°	55°
$P_{\mathrm{intake}}$ [bar-a]	1.26	1.4
$P_{\mathrm{inj}}$ [bar]	220	220
Swirl number	0.1	
Direct injector	Westport Fuel Systems $1^{\mathrm{st}}$ Generation HPDI	
Pilot fuel	Shell V-power (ULSD)	
Pilot fuel CN	>40 (CAN/CGSB-3.517)	
Primary fuel	Pipeline natural gas	
Primary fuel comp.	Typically > 96% CH_4_ (e.g. McTaggart-Cowan et al.^35,36^)	

**Figure 5.**
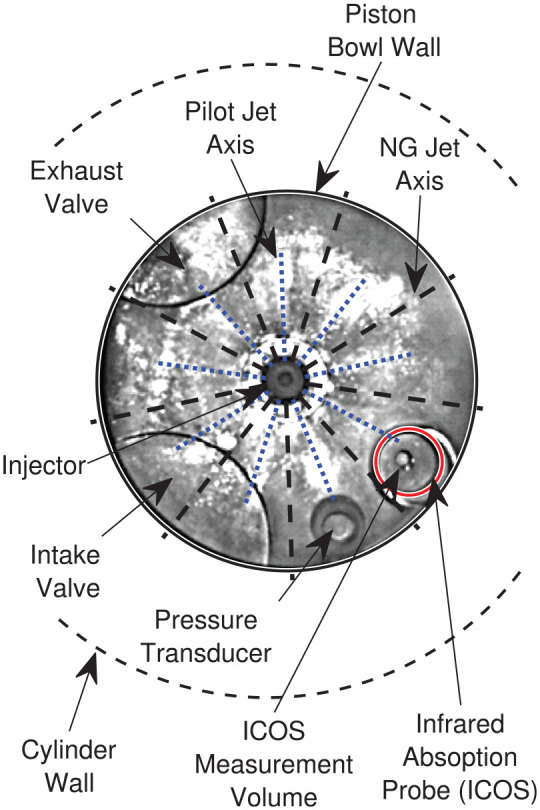
Camera view through Bowditch piston bowl.

The research engine facility is fitted with a first generation Westport Fuel Systems (WFS) High-Pressure Direct-Injection (HPDI) injector and commercial WFS dome-loaded self-relieving regulator (DLSR). A custom programmable engine control unit (ECU) and HPDI injector with independently actuated concentric needles allows arbitrary relative injection timing of the diesel and NG injections. The injector is mounted vertically and concentric to the piston bowl with 9 equally-spaced NG orifices and 9 pilot diesel orifices midway between each NG orifice (see Figure 5). Diesel rail pressure is controlled by the operator and the DLSR automatically maintains the NG rail pressure at 8 bar below the diesel rail pressure. Detailed characterization of the pipeline NG used for the primary fuel was out of scope in the current work. The effects of NG composition on the main PIDING combustion processes have been reported to be predominantly related to differences in fuel density (impacting injection duration) and PM emissions due to varying fractions of longer chain hydrocarbons.^36^ These observations have been made for non-premixed PIDING combustion, and it should be noted they may not apply to the wide range of stratified PIDING conditions considered in the current work.

A Bowditch piston with a flat-bottomed, cylindrical piston bowl housing a quartz window offset from the cylinder axis by 4 mm provides a 78 mm diameter optical access to the combustion chamber (see Figure 5). This piston bowl differs from the torroidal bowl in the thermodynamic piston and may affect jet impingement and fluid flow patterns, however AHRR measurement for all conditions indicated limited discrepancy in combustion behavior. The Bowditch piston also has a slightly lower geometric compression ratio, which requires adjustment of intake temperature and pressure to match $P_{\mathrm{cyl}}$ and estimated $T_{\mathrm{cyl}}$ at $\mathrm{SO}I_{\mathrm{pilot}}$ .

### Combustion imaging

Two imaging systems were used to simultaneously record: (i) OH*-CL at 310 nm and (ii) emission from PM at 700 nm. The imaging systems were synchronized to one another with a constant framerate of 15,000 Hz (≈0.4 CAD image temporal resolution) and focused to the horizontal midplane of the combustion chamber at TDC. All imaging is line-of-sight and although it is not possible to infer variations along the optical path, they provide qualitative indication of the position and intensity of reaction zones and soot clouds. Specifications of the imaging system hardware is provided in Table 2.

**Table 2.** Imaging system specifications.

Parameter	OH*-CL Imaging System
Camera	Photron SA-1 CMOS
Image intensifier	LaVision high speed IRO
Lens	Cerco 98 mm-f/2.8 UV
Aperture	f/2.8
Frame rate	15,000 Hz
Resolution	410 × 410
Narrow band-pass	310 nm CWL, 20 nm
Filter	FWHM
	700 nm Imaging System
Camera	Photron S-12 CMOS
Lens	60 mm-f/2.8 Micro-Nikkor
Aperture	f/2.8
Frame rate	15,000 Hz
Resolution	410 × 410
Narrow band-pass	700 nm CWL, 10 nm
filter	FWHM

OH*-CL measurements are used to analyze ignition, main combustion reaction zone structure and growth rates. The 700 nm images are used as an indicator of PM, with broadband incandescence from PM being the dominant emitter at 700 nm.^37^ The presence of PM has also been shown to significantly attenuate OH*-CL, which must be considered when analyzing OH*-CL for non-premixed combustion systems.^32^ Further detail on the analysis of OH*-CL images is provided in Appendix B.

Due to the wide range of combustion conditions imaged (i.e. non-premixed, partially-premixed, fully-premixed) the exposures were adjusted for each operating condition in order to maximize the dynamic range used on each camera sensor without incurring sensor saturation (exposures provided in Table 4). Where applicable, measurements of OH*-CL and PM image intensities are scaled by 1/exposure to allow direct comparison of different operating conditions.

### Local fuel concentration measurement

To characterize the fuel-air mixture development, an infrared absorption probe (LaVision ICOS) was used to measure the local CH_4_ concentration. The development and theory of the ICOS is described in detail elsewhere,^38^ and implementation of this instrument in the current experimental facility is described in previous work.^39^ A brief review of the theory and implementation are presented here.

The ICOS measures absorption of light sent via fiber optic cable from a quartz-tungsten-halogen lamp to a 20 mm^3^ measurement volume protruding from the cylinder head. Light introduced to the measurement volume is reflected by a mirrored surface, transmitted back to a second fiber optic cable and 3.4 μ m narrow band-pass filter before reaching the detector. This absorption band measures the C-H vibrational band, characteristic of hydrocarbon fuels, and is related to the fuel molar concentration within the measurement volume, $X_{\mathrm{fuel}}$ .

In the current work, the relative magnitude of $X_{\mathrm{fuel}}$ between operating conditions is analyzed. It is therefore permissible to not account for the sensitivity of the spectral absorption strength of the mixture, σ , to combustion chamber pressure and local fuel temperature, which are approximately equivalent throughout the compression stroke of all operating conditions considered in this work. To denote that this is a qualitative measurement, the relative fuel molar concentration is denoted as $X\prime_{\mathrm{fuel}}$ throughout the remainder of the discussion. The calculation of $X\prime_{\mathrm{fuel}}$ is developed in detail in Appendix A, and is summarized by equation (11):

(5) $$X\prime_{\mathrm{fuel}}(\theta )\alpha \left(\frac{\mathrm{In}\left(\frac{I\left(\theta \right)}{I_{o}}\right)}{P{\left(\theta \right)}^{\frac{1}{\gamma }}}\right)$$

Where I(θ) is the measured IR light intensity as a function of crank angle, $I_{o}$ is a reference light intensity measured each cycle, P(θ) is the measured cylinder pressure, and γ is the ratio of specific heats (assumed to be constant).

The ICOS provides a point measurement, therefore detailed observations of $X\prime_{\mathrm{fuel}}(\theta )$ are specific to the position of the ICOS measurement volume. As shown in Figure 5, the ICOS is located near the piston bowl wall (at 74% of bowl radius) and midway between two NG jet axes. Previous OH*-CL imaging of minimally-premixed PIDING combustion indicates this position is at a greater radius than the pilot ignition sites and is a suitable location for characterizing the NG mixture development prior to the partially-premixed combustion processes.^12^ Analysis of the phasing of CH_4_ consumption (i.e. rapid decrease of $X\prime_{\mathrm{fuel}}$ ) therefore provides a premixed combustion phasing measurement that is complementary to the line-of-sight OH*-CL imaging, but measured completely independently. The center of the measurement volume is positioned 9 mm below the firedeck (see Figure 4) to minimize the influence of combustion chamber walls on $X\prime_{\mathrm{fuel}}$ .

### Selected operating conditions

**Figure 6.**
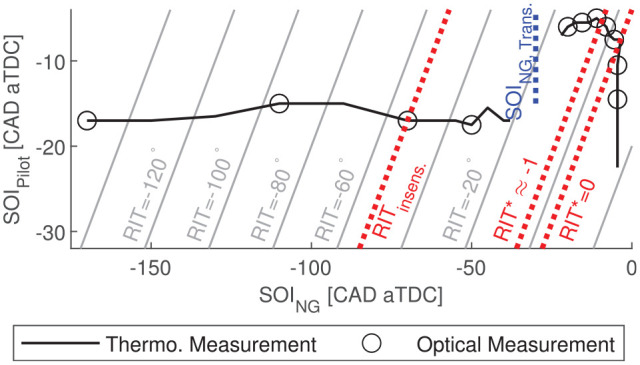
Operating conditions investigated with optical engine (circular markers). Previously investigated operating conditions from the thermodynamic engine configuration shown with solid line.^24^

The operating conditions considered in this investigation replicate a subset of recently published measurements collected using the thermodynamic engine configuration.^24^ There, a fine sweep of RIT was performed for −159°<RIT<+18° with $P_{\mathrm{inj}}=14,18,22$ MPa and ϕ=0.47,0.54,0.63,0.71 . Here, only $P_{\mathrm{inj}}=22$ MPa and ϕ=0.63 are considered for 11 RIT values. The 11 operating conditions were selected such that each regime of PIDING combustion has at least one measurement, with the exception of the transition NG jet impingement transition regime where combustion was too unstable to be measured in the optical engine. Engine operating parameters held constant across all considered operating conditions are presented in Table 3 and injection parameters are given in Table 4. $\mathrm{SO}I_{\mathrm{pilot}}$ and $\mathrm{SO}I_{\mathrm{NG}}$ given in Table 4 are the commanded injection timings. In all figures and analysis presented within this work the actual injection timings (i.e. including needle opening delay) are presented.^26^ The injector needle dynamics are also sensitive to cylinder pressure, so $\tau_{\mathrm{inj},\mathrm{NG}}$ was adjusted to maintain a constant $m_{\mathrm{NG}}$ for all $\mathrm{SO}I_{\mathrm{NG}}$ . In Figure 6, the selected operating conditions are presented in terms of $\mathrm{SO}I_{\mathrm{pilot}}$ and $\mathrm{SO}I_{\mathrm{NG}}$ , and are compared to the thermodynamic operating conditions previously investigated with $P_{\mathrm{inj}}=22$ MPa and ϕ=0.63 .

**Table 3.** Baseline operating conditions.

Operating parameter	Set-point
Speed [RPM]	1000
$T_{\mathrm{intake}}$ [°C]	55
$P_{\mathrm{intake}}$ [bar-a]	1.4
$m_{\mathrm{diesel}}$ [mg/cycle]	7±2
$m_{\mathrm{NG}}$ [mg/cycle]	92±3
NG energy fraction [%]	94
ϕ [-]	0.63
$P_{\mathrm{inj}}$ [bar]	220

**Table 4.** Summary of operating conditions investigated in optical engine. $\mathrm{SO}I_{\mathrm{NG}}$ and $\mathrm{SO}I_{\mathrm{pilot}}$ also presented in Figure 6.

Regime	RIT[CAD]	$\mathrm{SO}I_{\mathrm{pilot}}$ [CAD aTDC]	$\tau_{\mathrm{inj},\mathrm{pilot}}$ [ms]	$\mathrm{SO}I_{\mathrm{NG}}$ [CAD aTDC	$\tau_{\mathrm{inj},\mathrm{NG}}$ [ms]	Exposure(310 nm)[ μ s]	Exposure (700 nm) [ μ s]
$\mathrm{RI}T^{*}>0$	+10	−14.5	0.75	−4.5	1.27	18.0	1.7
$\mathrm{RI}T^{*}>0$	+6	−10.5	0.75	−4.5	1.27	18.0	1.6
$-1<\mathrm{RI}T^{*}<0$	+2	−7.5	0.75	−5.5	1.26	12.5	6.7
$-1<\mathrm{RI}T^{*}<0$	−2	−7.0	0.75	−9.0	1.24	7.0	2.0
$\mathrm{RI}T^{*}<-1$	−6	−6.0	0.75	−12.0	1.29	6.0	2.0
$\mathrm{RI}T^{*}<-1$	−10	−6.0	0.75	−16.0	1.31	6.5	12.5
$\mathrm{RI}T^{*}<-1$	−14	−7.0	0.75	−21.0	1.32	7.0	12.5
$\mathrm{RIT}>\mathrm{RI}T_{\mathrm{insens}.}$	−33	−19.0	0.75	−52.0	1.48	18.0	25.0
$\mathrm{RIT}\approx \mathrm{RI}T_{\mathrm{insens}.}$	−53	−17.0	0.75	−71.0	1.50	9.0	25.0
$\mathrm{RIT}<\mathrm{RI}T_{\mathrm{insens}.}$	−95	−17.0	0.75	−110.0	1.50	10.0	25.0
$\mathrm{RIT}<\mathrm{RI}T_{\mathrm{insens}.}$	−153	−18.0	0.75	−171.0	1.50	12.0	25.0

The engine was operated in a skip-firing mode consisting of 3 consecutive fired cycles followed by 17 motored cycles (no combustion) to allow the window to cool. Images were recorded on the $3^{\mathrm{rd}}$ fired cycle as there were no significant differences in AHRR between the third and subsequent fired cycles. The images, AHRR, and ICOS measurements presented in the current work are ensemble averaged from a set of 15 skip-firing sequences (i.e. 15 imaged cycles) unless explicitly indicated to be single-cycle measurements. Using an intake by-pass valve (see Figure 4), the intake air system was pre-conditioned to the desired temperature and pressure prior to every test. This reduced variability in the intake charge conditions between tests and improved repeatability of measurements.

## Characterization of regimes of PIDING combustion

In this work, characterization of the NG mixture development and the resulting features of the combustion structure(s) are investigated to refine descriptions of the regimes of PIDING combustion previously proposed based on emissions and AHRR analysis. Local relative fuel-air ratio, $X\prime_{\mathrm{fuel}}$ , for non-reacting cases (no pilot ignition) is assessed to qualitatively characterize the NG mixture development with respect to RIT and NG premixing time, $\tau_{\mathrm{NG}}$ . Subsequently, $X\prime_{\mathrm{fuel}}$ for reacting conditions is compared to in-cylinder imaging and AHRR to characterize the reaction zone structures and relative phasing of premixed fuel consumption for each regime of PIDING combustion. To distinguish characteristics of each regime of PIDING combustion, the discussion compares adjacent domains of RIT :

1. Minimally-premixed → variable-premixed
2. Variable-premixed → stratified-premixed (late-cycle)
3. Stratified-premixed (early-cycle) → RIT-insensitive premixed

To describe the spectrum of premixed PIDING combustion regimes, a summary of $X\prime_{\mathrm{fuel}}$ characteristics, AHRR features, and reaction zone structures is presented as a function of RIT for all regimes of PIDING combustion.

### Non-reacting NG mixture development

The NG mixture stratification is a key parameter influencing ignition, main combustion, and emissions behavior for all regimes of PIDING combustion. Following direct injection of the NG, complex fluid mixing processes will cause the fuel-air mixture to develop from a highly stratified state (i.e. pure fuel in the core of the NG jet) toward a fully-developed homogeneous state. The transient fuel-air mixture states are a function of the NG premixing time, $\tau_{\mathrm{NG}}$ , and the turbulent flow field of the combustion chamber. The NG premixing time is readily controlled by RIT , however the flow field is a function of a multitude of parameters many of which are also time-varying (e.g. chamber geometry, turbulent kinetic energy, etc.).

**Figure 7.**
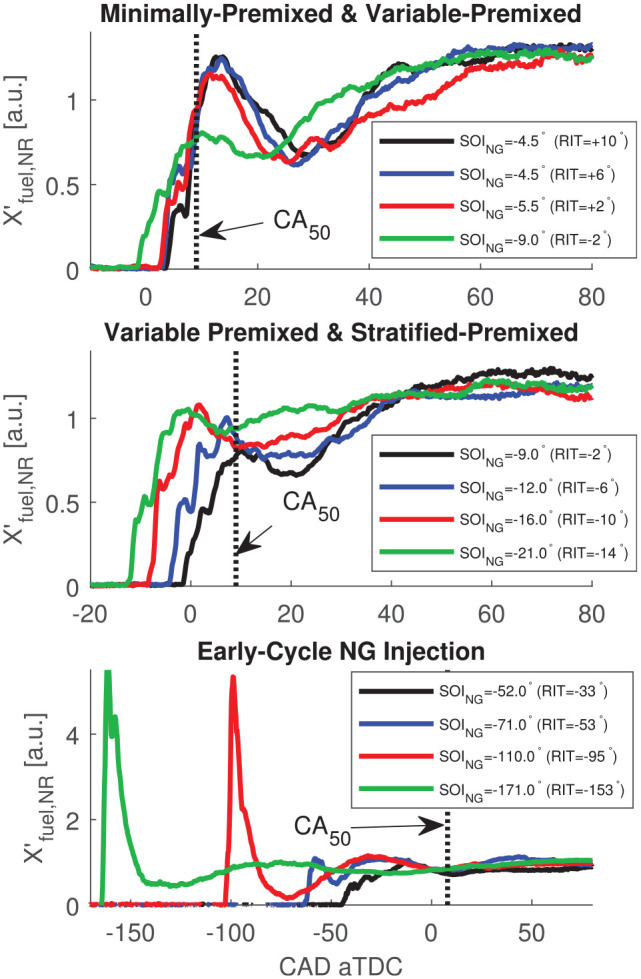
Comparison of non-fired (i.e. NG injection only) relative equivalence ratio, $X\prime_{\mathrm{fuel},\mathrm{NR}}$ for all PIDING operating conditions.

In Figure 7, $X\prime_{\mathrm{fuel}}$ for non-reacting operation, $X\prime_{\mathrm{fuel},\mathrm{NR}}$ , is presented to characterize fuel-air mixing for the considered regimes of PIDING combustion. Non-reacting engine operation was performed by removing the pilot injection (i.e. the ignition source) and maintaining all other measurement parameters equivalent to the corresponding reacting condition. Figure 7 divides the measurements into the three ranges of RIT , which are also used to structure subsequent discussion of the corresponding reacting cases. As a point measurement, $X\prime_{\mathrm{fuel}}$ is sensitive to turbulent advection of fuel, which results in high cyclic variability of $X\prime_{\mathrm{fuel}}$ shortly after $\mathrm{SO}I_{\mathrm{NG}}$ when the NG distribution is most heterogeneous. For the relatively small sample sizes discussed in this work (15 repeated measurements), this cyclic variability can impact the ensemble averaged $X\prime_{\mathrm{fuel}}$ shortly after $\mathrm{SO}I_{\mathrm{NG}}$ (e.g. small difference between two operating conditions with $\mathrm{SO}I_{\mathrm{NG}}=+4.5{}^{\circ}$ in Figure 7).

For all non-fired operating conditions shown in Figure 7, $X\prime_{\mathrm{fuel},N\;R}$ is a strong, non-monotonic function of the NG premixing time (i.e. CAD after $\mathrm{SO}I_{\mathrm{NG}}$ ) for approximately 60–70 CAD (≈10–12 ms) after $\mathrm{SO}I_{\mathrm{NG}}$ before a homogeneous mixture is indicated by $d/\mathrm{dt}(X\prime_{\mathrm{fuel},\mathrm{NR}})\approx 0$ . Features of the development of $X\prime_{\mathrm{fuel},N\;R}$ are also sensitive to RIT for conditions where $\mathrm{SO}I_{\mathrm{NG}}$ is varied (all RIT<−2° , see Figure 6). To highlight the difference in NG mixture development as it pertains to combustion, approximate phasing of 50% indicated heat release, $CA_{50}$ , is also shown in Figure 7.

For all conditions with late-cycle injections (top and middle plot of Figure 7), NG stratification has not fully-developed by the time of combustion. The peak $X\prime_{\mathrm{fuel},N\;R}$ occurs after $C\;A_{50}$ for RIT≥2 CAD ( $\mathrm{RI}T^{*}\geq -0.3$ ), but prior to $CA_{50}$ for RIT≤−2 CAD ( $\mathrm{RI}T^{*}\leq -0.8$ ). $X\prime_{\mathrm{fuel},N\;R}$ in the bottom plot of Figure 7 indicates fully-developed homogeneous fuel-air mixtures at the time of combustion are likely for very early $\mathrm{SO}I_{\mathrm{NG}}$ , but may not have developed for RIT=−33°,−53° .

The development of $X\prime_{\mathrm{fuel},N\;R}$ for early-cycle NG injections (bottom plot of Figure 7) is distinct from that of late-cycle injections due to very different chamber conditions (i.e. chamber geometry, charge density, and injection pressure ratio). The very high initial $X\prime_{\mathrm{fuel},N\;R}$ in the bottom plot of Figure 7 is a result of the NG jet passing the ICOS measurement volume while there is low charge density for very early $\mathrm{SO}I_{\mathrm{NG}}$ .

While the $X\prime_{\mathrm{fuel},N\;R}$ behavior for each operating condition is particular to the location of the fuel concentration measurement volume, the observed behavior demonstrates that $\mathrm{SO}I_{\mathrm{NG}}$ and the subsequent interaction of the NG injection with the cylinder flow field has significant implications for the development of the NG-air mixture.

### Variable premixed fraction regime

For PIDING combustion in the minimally-premixed and variable premixed fraction regimes, late $\mathrm{SO}I_{\mathrm{NG}}$ produces heterogeneous fuel-air mixtures. In the minimally-premixed regime, $\tau_{\mathrm{NG}}$ has a minimum value and $\tau_{\mathrm{NG}}\neq f(\mathrm{RIT})$ which indicates that the premixed fraction of NG, $f_{\mathrm{premix}}$ is also at a minimum and NG stratification is therefore at a maximum. Combustion transitions to the variable premixed fraction regime when RIT is reduced below $\mathrm{RI}T^{*}=0$ and $\tau_{\mathrm{NG}}$ begins to increase.^24^

**Figure 8.**
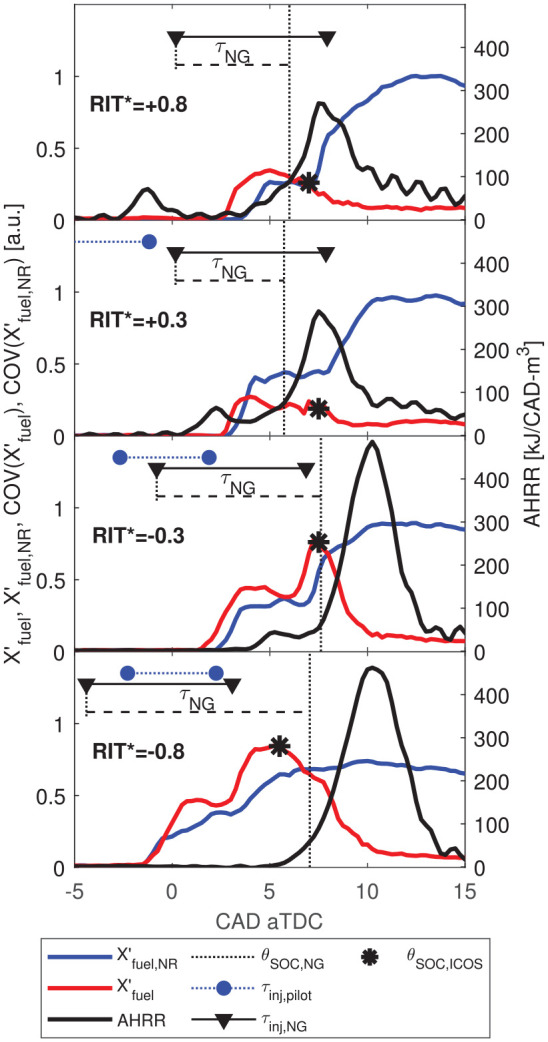
Comparison of AHRR, $X\prime_{\mathrm{fuel}}$ , and $X\prime_{\mathrm{fuel},\mathrm{NR}}$ to assess relative phasing of the start of NG combustion, $\theta_{\mathrm{SOC},\mathrm{NG}}$ and fuel consumption at the ICOS, $\theta_{\mathrm{SOC},\mathrm{ICOS}}$ (*) for minimally-premixed ( $\mathrm{RI}T^{*}>0$ ) and variable premixed fraction ( $-1<\mathrm{RI}T^{*}<0$ ) operating conditions. Ensemble averaged quantities shown.

In Figure 8, $X\prime_{\mathrm{fuel}}$ for reacting and non-reacting operation ( $X\prime_{\mathrm{fuel}}$ and $X\prime_{\mathrm{fuel},\mathrm{NR}}$ , respectively) are compared to AHRR for minimally-premixed and variable-premixed fraction operating conditions. For $\mathrm{RI}T^{*}>0$ , $X\prime_{\mathrm{fuel}}$ increases in the interval between the pilot AHRR and the start of the main NG combustion ( $\theta_{\mathrm{SOC},\mathrm{NG}}$ ) indicating some mass of NG penetrates past the pilot ignition regions and premixes. However, the maximum $X\prime_{\mathrm{fuel}}$ is limited to well below the peak of $X\prime_{\mathrm{fuel},\mathrm{NR}}$ because a significant mass of NG is consumed in non-premixed combustion prior to reaching the ICOS measurement volume. For $\mathrm{RI}T^{*}<0$ , decreasing $\mathrm{RI}T^{*}$ increases $\tau_{\mathrm{NG}}$ and therefore a greater mass of NG premixes, which is measured as an increased peak $X\prime_{\mathrm{fuel}}$ in Figure 8.

The start of premixed NG conversion at the ICOS is indicated by a sharp drop in $X\prime_{\mathrm{fuel}}$ , which is denoted $\theta_{\mathrm{SOC},\mathrm{ICOS}}$ and indicated with an (*) in Figure 8. For all conditions shown in Figure 8, $\theta_{\mathrm{SOC},\mathrm{ICOS}}$ precedes peak $X\prime_{\mathrm{fuel},\mathrm{NR}}$ because $\tau_{\mathrm{NG}}$ is insufficient for the complete mass of NG to premix (i.e. $f_{premix}<100\%$ ) for $\mathrm{RI}T^{*}>-0.8$ . For all conditions shown in Figure 8, $X\prime_{\mathrm{fuel}}$ diverges from $X\prime_{\mathrm{fuel},\mathrm{NR}}$ prior to $\theta_{\mathrm{SOC},\mathrm{ICOS}}$ , indicating some influence of the pilot injection on the NG concentration measurement. This may be due to pressure and temperature effects on the absorption strength coefficient ( σ ), pilot injections modifying the fluid mixing field in the combustion chamber, and/or injector dynamics.

**Figure 9.**
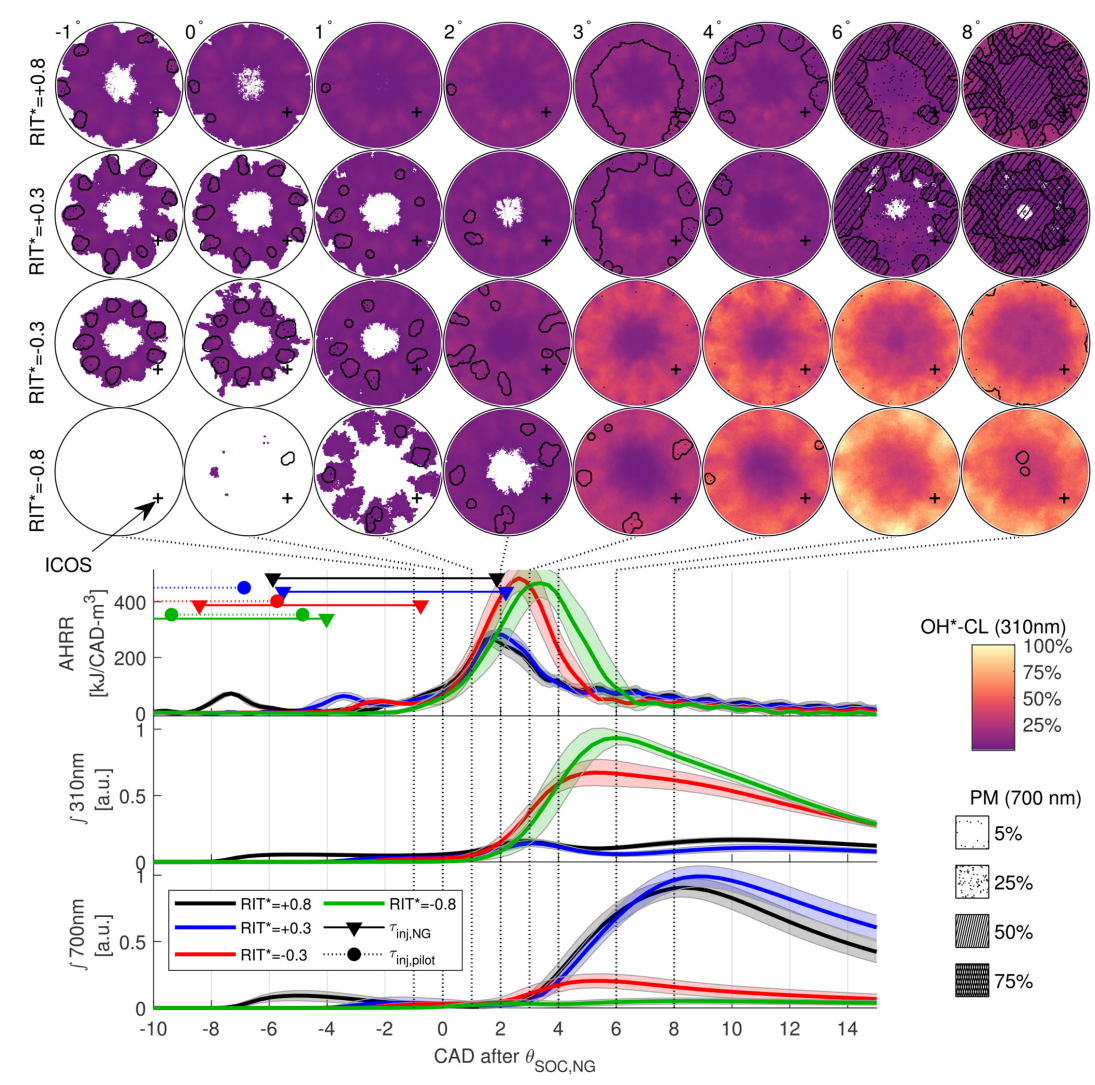
Comparison of ensemble averaged images of OH*-CL (310 nm) and PM (700 nm) with AHRR, ∫310 nm, and ∫700 nm for minimally-premixed ( $\mathrm{RI}T^{*}>0$ ) and variable premixed fraction ( $\mathrm{RI}T^{*}<0$ ) operating conditions. Note all temporal phasing is relative to $\theta_{\mathrm{SOC},\mathrm{NG}}$ . Shaded regions in AHRR, ∫310 nm and ∫700 nm indicate cycle to cycle standard deviation of the respective measurement.

To investigate the spatial distribution of the reaction zones for minimally-premixed and variable-premixed fraction PIDING combustion, Figure 9 presents in-cylinder imaging for $-0.8<\mathrm{RI}T^{*}<+0.8$ . Images in Figure 9 present the ensemble averaged images recorded at 310 nm (OH*-CL) with 700 nm (nominally PM) images overlaid. The overlay of the 700 nm images as a hatch is used to indicate that the presence of PM contributes to significant attenuation of the OH*-CL signal; locations where there is a strong PM signal, the local magnitude of the measured OH*-CL intensity cannot be reliably interpreted or compared to other regions.^12,32^ The images for each operating condition are compared to the AHRR, and integrated light intensity ( ∫310 nm and ∫700 nm), which are phased relative to $\theta_{\mathrm{SOC},\mathrm{NG}}$ . The shaded regions for AHRR, ∫310 nm, and ∫700 nm indicate the standard deviation of the measurement as a function of crank angle.

The ensemble averaged images in Figure 9 demonstrate significantly different reaction zone structures for minimally-premixed ( $\mathrm{RI}T^{*}>0$ ) and variable premixed fraction ( $\mathrm{RI}T^{*}<0$ ) combustion. For $\mathrm{RI}T^{*}>0$ , the non-premixed NG jet structures are visible in the OH*-CL from 0°−4° after $\theta_{\mathrm{SOC},\mathrm{NG}}$ . The non-premixed combustion results in a strong PM signal developing near the piston bowl wall after peak AHRR, which agrees with pyrometric imaging of similar PIDING operating conditions.^32^ For $\mathrm{RI}T^{*}>0$ in Figure 9, ∫310 nm and ∫700 nm are also very similar in phasing and magnitude, indicating that the constant $\tau_{\mathrm{NG}}$ for all minimally-premixed PIDING combustion modes produces nominally the same main combustion processes.

A significantly different main combustion process is observed for variable premixed fraction combustion ( $\mathrm{RI}T^{*}<0$ in Figure 9), where $f_{\mathrm{premix}}$ increases with decreasing $\mathrm{RI}T^{*}$ . The increased $f_{\mathrm{premix}}$ reduces the locally-rich non-premixed combustion, which results in significantly reduced PM in the images and low ∫700 nm for $\mathrm{RI}T^{*}=-0.3,-0.8$ . The OH*-CL images (310 nm) and peak AHRR for $\mathrm{RI}T^{*}<0$ indicate that a more rapid fuel conversion process near the piston bowl wall becomes dominant as $\mathrm{RI}T^{*}$ is reduced from the minimally-premixed to variable-premixed combustion regime. At $\theta_{\mathrm{SOC},\mathrm{NG}}$ , the OH*-CL for $\mathrm{RI}T^{*}<0$ is significantly reduced relative to conditions with $\mathrm{RI}T^{*}>0$ , due to pilot quenching by the NG jets.^24^ Despite the increased $\tau_{\mathrm{NG}}$ for the variable premixed fraction conditions ( $\mathrm{RI}T^{*}<0$ ), the OH*-CL in the center of the combustion chamber remains weak throughout the cycle. This is an indication of the incomplete premixing of the NG, which is also indicated by $X\prime_{\mathrm{fuel}}$ in Figures 7 and 8 for all conditions shown in Figure 9.

**Figure 10.**
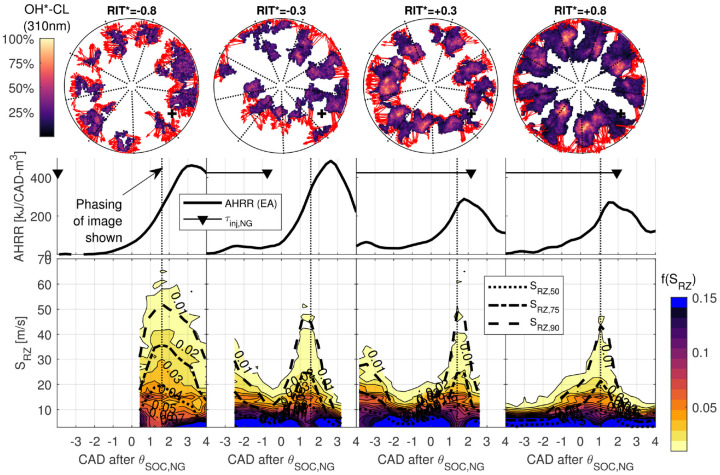
Distribution of local reaction zone speed, $S_{\mathrm{RZ}}$ , presented as a fraction, $f(S_{\mathrm{RZ}})$ , of all local measurements at each recorded frame. 50^*th*^, 75^*th*^, and 90^*th*^ percentiles of $S_{\mathrm{RZ}}$ shown ( $S_{\mathrm{RZ},50}$ , $S_{\mathrm{RZ},75}$ , $S_{\mathrm{RZ},90}$ ). Representative single-cycle OH*-CL images (image phasing indicated by dotted line) with red vectors indicating the measured displacement of reaction zone boundaries used to calculate $S_{\mathrm{RZ}}$ .

In Figure 10, the local reaction zone speed, $S_{\mathrm{RZ}}$ , is presented to investigate differences in the fuel conversion processes for $\mathrm{RI}T^{*}\geq -0.8$ . $S_{\mathrm{RZ}}$ is evaluated by applying a pixel intensity threshold to single-cycle OH*-CL images of PIDING combustion and measuring the distance between the boundaries of thresholded images in consecutive frames for every point on the perimeter of the first thresholded image. This method has previously been described in detail for characterization of flame propagation speeds in premixed dual-fuel combustion.^40^ While $S_{\mathrm{RZ}}$ is related to the reaction zone growth rate, quantitative analysis of this measurement is limited by the line-of-sight imaging of OH*-CL used here.

In Figure 10, the distribution of $S_{\mathrm{RZ}}$ is presented as a probability density of $S_{\mathrm{RZ}}$ , $f(S_{\mathrm{RZ}})$ (binned in intervals of 2 m/s). For each operating condition, a representative single-cycle OH*-CL image is presented with red vectors indicating the local $S_{\mathrm{RZ}}$ . For conditions with $\mathrm{RI}T^{*}\geq -0.3,$ an early peak in $S_{\mathrm{RZ}}$ during pilot ignition (distributed auto-ignition) is followed by a second larger peak during the main premixed NG combustion heat release peak. The phasing and magnitudes of the peaks in $S_{\mathrm{RZ}}$ for $\mathrm{RI}T^{*}\geq -0.3$ agree with previous measurements of PIDING combustion for $\mathrm{RI}T^{*}>0$ .^12^ However, for $\mathrm{RI}T^{*}=-0.8$ there is only a single peak in the $S_{\mathrm{RZ}}$ . This suggests pilot and NG ignition processes occur simultaneously, rather than sequentially.

Despite the significantly higher peak AHRR and increased $f_{\mathrm{premix}}$ for $\mathrm{RI}T^{*}=-0.3$ compared to $\mathrm{RI}T^{*}>0$ (see Figure 8), the peak $S_{\mathrm{RZ}}$ in Figure 10 are very similar for these conditions. The single-cycle OH*-CL images for $\mathrm{RI}T^{*}\geq -0.3$ show that the larger $S_{\mathrm{RZ}}$ vectors are predominantly oriented radially-outward, which may indicate that the high $S_{\mathrm{RZ}}$ observed during the premixed NG combustion for $\mathrm{RI}T^{*}\geq -0.3$ is generated by the NG injection momentum. For $\mathrm{RI}T^{*}=-0.8,$ a significant increase in the peak $S_{\mathrm{RZ}}$ and more isotropic orientation of the $S_{\mathrm{RZ}}$ vectors indicates different processes drive reaction zone growth for $\mathrm{RI}T^{*}=-0.8$ compared to $\mathrm{RI}T^{*}\geq -0.3$ .

### Late-cycle stratified-premixed regime

Late-cycle stratified-premixed PIDING combustion is defined by NG injection impingement within the piston bowl and the entire mass of NG premixing prior to ignition (i.e. $f_{premix}=100\%$ ). The exact RIT at which $f_{premix}=100\%$ occurs is challenging to directly measure, however the phasing of peak $X\prime_{\mathrm{fuel},\mathrm{NR}}$ (Figure 7) suggests that $\mathrm{RI}T^{*}\approx -1$ (see equation (4)) is a reasonable estimate. Although $f_{premix}=100\%$ , late-cycle NG injections do not produce $\tau_{\mathrm{NG}}$ sufficiently long for a uniform mixture distribution (i.e. steady state $X\prime_{\mathrm{fuel}}$ ) to develop (see Figure 7). It is therefore expected that NG stratification will be an important factor in the ignition, main combustion, and emissions performance in the stratified-premixed combustion regime.

**Figure 11.**
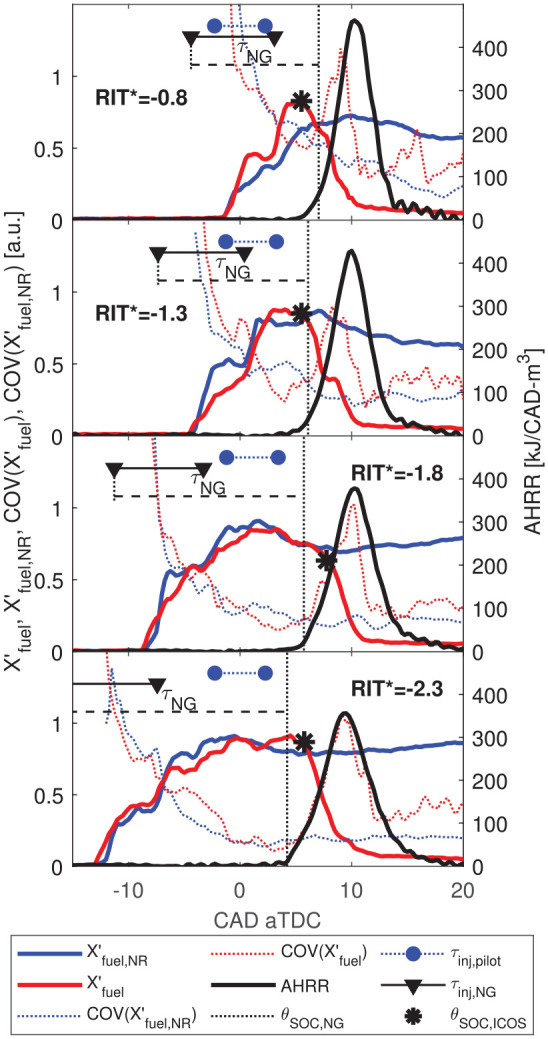
Comparison of AHRR, $X\prime_{\mathrm{fuel}}$ , and COV $\left(X\prime_{\mathrm{fuel},N\;R}\right)$ to assess relative phasing of $\theta_{\mathrm{SOC},\mathrm{NG}}$ and $\theta_{\mathrm{SOC},\mathrm{ICOS}}$ and the NG mixture variability for variable premixed fraction ( $-1\leq \mathrm{RI}T^{*}<0$ ) and stratified-premixed ( $\mathrm{RI}T^{*}<-1$ ) operating conditions. Ensemble averaged quantities shown.

In Figure 11, $X\prime_{\mathrm{fuel}}$ and $X\prime_{\mathrm{fuel},\mathrm{NR}}$ are compared to AHRR for −2.3≤RIT≤−0.8 . COV of $X\prime_{\mathrm{fuel}}$ is also presented in Figure 11 to describe the cyclic variability of the mixture development processes. For $\mathrm{RI}T^{*}=-0.8,-1.3$ , $\theta_{\mathrm{SOC},\mathrm{ICOS}}$ precedes peak $X\prime_{\mathrm{fuel},N\;R}$ indicating pilot ignition is occurring within premixed NG near the ICOS probe. For $\mathrm{RI}T^{*}=-0.8,-1.3$ , $X\prime_{\mathrm{fuel}}$ deviates from $X\prime_{\mathrm{fuel},\mathrm{NR}}$ prior to ignition, which indicates systematic differences in the NG mixing processes for the reacting and non-reacting NG injections. Conversely, for $\mathrm{RI}T^{*}=-1.8,-2.3,$ where the NG and pilot injections do not overlap (i.e. $\mathrm{SO}I_{\mathrm{pilot}}>\mathrm{EO}I_{\mathrm{NG}}$ ), the deviation of $X\prime_{\mathrm{fuel}}$ and $X\prime_{\mathrm{fuel},\mathrm{NR}}$ prior to ignition is significantly reduced. This suggests that the pilot injection impacts the NG mixing processes due to injector dynamics and/or in-cylinder mixing processes when pilot and NG injections overlap temporally.

Increased $\tau_{\mathrm{NG}}$ for $\mathrm{RI}T^{*}=-1.8,-2.3$ results in comparatively steady $X\prime_{\mathrm{fuel},N\;R}$ (relative to $\mathrm{RI}T^{*}\geq -1.3$ ) and low COV( $X\prime_{\mathrm{fuel}}$ , $X\prime_{\mathrm{fuel},N\;R}$ ) evaluated at $\theta_{\mathrm{SOC},\mathrm{NG}}$ (COV $(X\prime_{\mathrm{fuel}}){|}_{\theta \mathrm{SOC},N\;G}$ ). This indicates decreasing CCV of the NG mixing processes with increasing $\tau_{\mathrm{NG}}$ , which was previously observed as decreasing COV of indicated mean effective pressure for the same range of $\mathrm{RI}T^{*}$ .^24^

**Figure 12.**
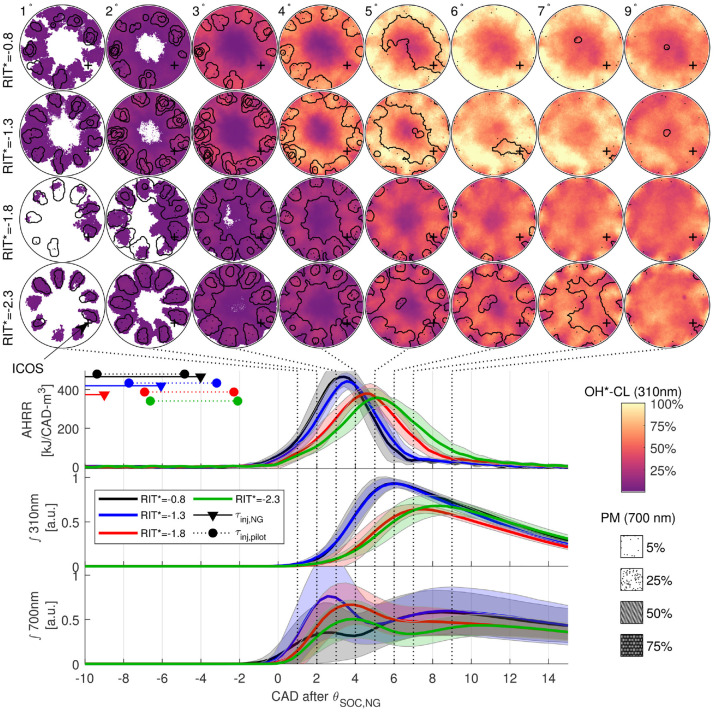
Comparison of ensemble averaged images of OH*-CL (310 nm) and PM (700 nm) with AHRR, ∫310 nm, and ∫700 nm for variable premixed fraction ( $-1\leq \mathrm{RI}T^{*}<0$ ) and late-cycle stratified-premixed fraction ( $\mathrm{RI}T^{*}<-1$ ) operating conditions. Note that all temporal phasing is relative to $\theta_{\mathrm{SOC},\mathrm{NG}}$ . Shaded regions in AHRR, ∫310 nm and ∫700 nm indicate cycle to cycle standard deviation of the respective measurement.

OH*-CL (310 nm) and PM (700 nm) imaging is compared for $\mathrm{RI}T^{*}=-0.8,-1.3,-1.8,-2.3$ in Figure 12. The AHRR, reaction zone structures (OH*-CL), and PM structures change significantly between −1.8<RIT<−1.3 with higher peak AHRR and ∫310 nm for $\mathrm{RI}T^{*}\geq -1.3$ . For $\mathrm{RI}T^{*}\geq -1.3$ , the regions of highest intensity reactions (indicated by OH*-CL) form a ring around the bowl wall, where premixed NG combustion has been observed for minimally-premixed and variable premixed fraction combustion.^12^ With increasing $\tau_{\mathrm{NG}}$ (i.e. increasingly negative $\mathrm{RI}T^{*}$ ) the OH*-CL intensity in this ring diminishes significantly near peak AHRR ( 3°−6° after $\theta_{\mathrm{SOC},\mathrm{NG}}$ in Figure 12), which is accompanied by a significant decrease of peak AHRR. The decrease in OH*-CL is most significant between $\mathrm{RI}T^{*}=-1.3$ and $\mathrm{RI}T^{*}=-1.8$ , which coincides with the $\mathrm{RI}T^{*}$ for which $X\prime_{\mathrm{fuel},N\;R}$ decreases prior to $\theta_{\mathrm{SOC},\mathrm{ICOS}}$ , indicating increased fuel mixing away from the bowl wall.

In Figure 12, the ensemble averaged images for θ>3° after $\theta_{\mathrm{SOC},\mathrm{NG}}$ show increasing definition of distinct reaction zones as $\mathrm{RI}T^{*}$ is reduced (i.e. 9 distinct reaction zones for each NG jet become more clear for more negative $\mathrm{RI}T^{*}$ ). This indicates that there is lower CCV in the reaction zone structure and location as $\tau_{\mathrm{NG}}$ increases for the late-cycle stratified-premixed regime. This may be a result of the decreasing CCV of NG mixture formation indicated by decreasing COV( $X\prime_{\mathrm{fuel}}){|}_{\theta \mathrm{SOC},\mathrm{NG}}$ (see Figure 11). The relatively high variability in the reaction zone structures for $\mathrm{RI}T^{*}=-0.8,-1.3$ may also be a result of pilot quenching by the NG jet, which was previously measured for these conditions.^24^

For all conditions shown in Figure 12, PM indicated by 700 nm imaging is significantly lower than for the minimally-premixed conditions shown in Figure 9 (maximum 700 nm signal is a factor of 11 greater in Figure 9 than in Figure 12). For the late-cycle stratified conditions shown, the most intense PM is observed before the peak AHRR (2–3 CAD after $\theta_{\mathrm{SOC},\mathrm{NG}}$ in Figure 12). This contrasts observations of non-premixed and variable premixed fraction combustion regimes where peak PM results from the main NG combustion process following peak AHRR (see Figure 9).^32^ For the stratified-premixed conditions, the peak PM signal is also localized in the 9 pilot ignition regions, indicating the pilot ignition process is more significant for PM production than the premixed NG combustion process for these operating conditions. For $\mathrm{RI}T^{*}=-0.8$ where pilot quenching is most significant,^24^ the initial peak in the ∫700 nm is further reduced, indicating that PM produced in the pilot reactions is mitigated by the quenching.

**Figure 13.**
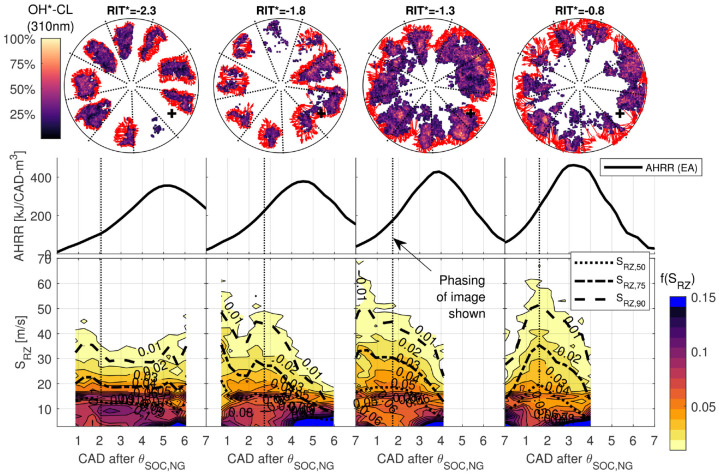
Distribution of local reaction zone speed, $S_{\mathrm{RZ}}$ , presented as a fraction, $f(S_{\mathrm{RZ}})$ , of all local measurements at each recorded frame. 50^*th*^, 75^*th*^, and 90^*th*^ percentiles of $S_{\mathrm{RZ}}$ shown ( $S_{\mathrm{RZ},50}$ , $S_{\mathrm{RZ},75}$ , $S_{\mathrm{RZ},90}$ ). Representative single-cycle OH*-CL images (image phasing indicated by dotted line) with red vectors indicating the measured displacement of reaction zone boundaries used to calculate $S_{\mathrm{RZ}}$ .

In Figure 13, the distribution of reaction zone speeds, $S_{\mathrm{RZ}}$ , is compared for $\mathrm{RI}T^{*}=-0.8,-1.3,-1.8,-2.3$ . For $\mathrm{RI}T^{*}=-0.8$ , where there is measurable quenching of the pilot by the NG jets,^24^ there is a single peak in the $S_{\mathrm{RZ}}$ distribution. In contrast, for $\mathrm{RI}T^{*}\leq -1.3$ (no measurable pilot quenching) there is a peak in $S_{\mathrm{RZ}}$ due to pilot auto-ignition followed by high $S_{\mathrm{RZ}}$ around 2 CAD after $\theta_{\mathrm{SOC},\mathrm{NG}}$ . With increasing $\tau_{\mathrm{NG}}$ (i.e. decreasing $\mathrm{RI}T^{*}$ ), peak AHRR and peak $S_{\mathrm{RZ}}$ after pilot-ignition decrease. This trend of decreasing $S_{\mathrm{RZ}}$ may result from: decreasing local NG concentration near the bowl wall (where the premixed combustion tends to be most prominent, see Figure 12), decay of injection-generated turbulence, and/or reduced entrainment of the reactive pilot fuel from pilot quenching.

The single-cycle images presented in Figure 13 indicate a significant change in reaction zone structure for $\mathrm{RI}T^{*}\geq -1.3$ and $\mathrm{RI}T^{*}\leq -1.8$ . For $\mathrm{RI}T^{*}=-0.8,-1.3$ premixed NG combustion initiates in a large reaction zone volume located close to the bowl wall (where $X\prime_{\mathrm{fuel}}$ is measured). In contrast, for $\mathrm{RI}T^{*}=-1.8,-2.3$ , $\mathrm{EO}I_{\mathrm{NG}}$ is early enough that pilot quenching does not occur, so pilot reactants remain near the center of the combustion chamber. Ignition therefore occurs closer to the center of the chamber and the reaction zone must propagate outward through more thoroughly premixed NG.

### Early-cycle combustion regimes

With early-cycle $\mathrm{SO}I_{\mathrm{NG}}$ , NG jet impingement outside the piston bowl and long $\tau_{\mathrm{NG}}$ produce more homogeneous mixture properties prior to the start of combustion relative to late-cycle combustion regimes. Analysis of $X\prime_{\mathrm{fuel},N\;R}$ in Figure 7 indicates that a steady-state NG concentration distribution is developed by typical $C\;A_{50}$ for RIT≤−53° . This coincides with a marked decrease in the sensitivity of emissions to RIT that was previously identified at $\mathrm{RI}T_{\mathrm{insens}.}=-53{}^{\circ}$ .^24^ However, for $\mathrm{RIT}<\mathrm{RI}T_{\mathrm{insens}.}$ the AHRR shape was still sensitive to variation of RIT , indicating parameters other than fuel concentration distribution were significant for combustion processes.

**Figure 14.**
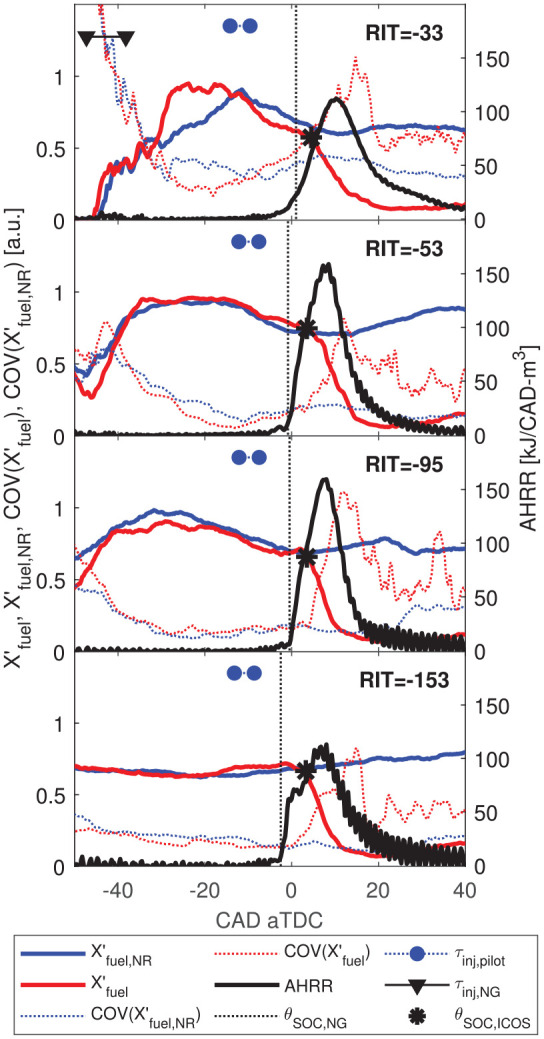
Comparison of AHRR, $X\prime_{\mathrm{fuel}}$ , and COV $\left(X\prime_{\mathrm{fuel}}\right)$ to assess relative phasing of $\theta_{\mathrm{SOC},\mathrm{NG}}$ and $\theta_{\mathrm{SOC},\mathrm{ICOS}}$ and the NG mixture variability for early-cycle PIDING combustion conditions. Ensemble averaged quantities shown.

The development of $X\prime_{\mathrm{fuel}}$ for early-cycle PIDING combustion with $\mathrm{RIT}>\mathrm{RI}T_{\mathrm{insens}.}$ , $\mathrm{RIT}\approx \mathrm{RI}T_{\mathrm{insens}.}$ , and $\mathrm{RIT}<\mathrm{RI}T_{\mathrm{insens}.}$ is shown in Figure 14. For $\mathrm{RIT}\approx \mathrm{RI}T_{\mathrm{insens}.}$ ( RIT=−53° ), COV( $X\prime_{\mathrm{fuel}}$ ) reaches a minimum value shortly prior to the start of combustion ( $\theta_{\mathrm{SOC},\mathrm{NG}}$ ), which is approximately equal to the corresponding COV( $X\prime_{\mathrm{fuel}}$ ) for the operating conditions with much longer $\tau_{\mathrm{NG}}$ ( RIT=−95°,−153° ). This indicates $\mathrm{RI}T_{\mathrm{insens}.}$ is a reasonable estimate of the injection phasing required for CCV of the NG mixing processes to reach steady state prior to combustion.

For $\mathrm{RIT}>\mathrm{RI}T_{\mathrm{insens}.}$ ( RIT=−33° ), COV( $X\prime_{\mathrm{fuel}}){|}_{\theta \mathrm{SOC},\mathrm{NG}}$ in Figure 14 is relatively high, and unlike all other operating conditions (both late- and early-cycle) is increasing rather than decreasing prior to the start of combustion. This unique mixture development behavior for RIT=−33° likely results from impingement of the NG jet near the piston bowl edge, which causes a highly variable $X\prime_{\mathrm{fuel}}$ and AHRR. Due to the high CCV of both $X\prime_{\mathrm{fuel}}$ and AHRR, the ensemble average of both these quantities is not representative of the majority of measured cycles.

For all early-cycle operating conditions in Figure 14, $X\prime_{\mathrm{fuel}}$ begins to increase after the combustion event starting at approximately 20° aTDC. This likely indicates significant unburned fuel from quench and crevice volumes in the combustion chamber entering the ICOS measurement volume. $X\prime_{\mathrm{fuel}}$ measured subsequent to the combustion event (average $X\prime_{\mathrm{fuel}}$ from 30 to 90 CAD aTDC), $X\prime_{\mathrm{fuel},\mathrm{post}}$ , correlates with exhaust CH_4_ emissions measured using the thermodynamic engine (see Appendix C).^24^

**Figure 15.**
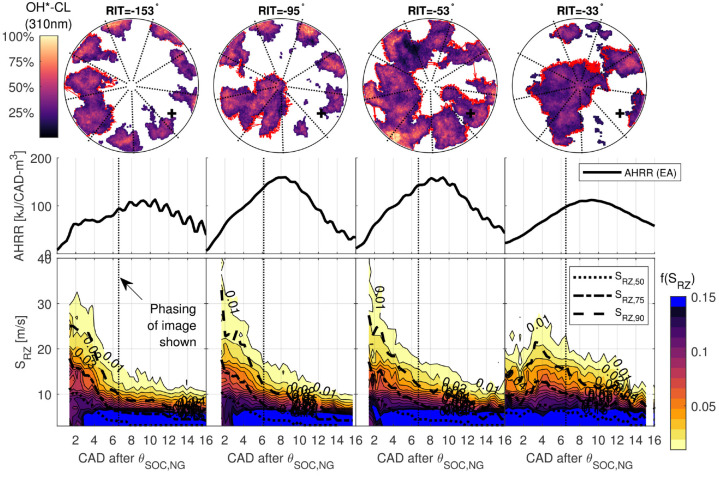
Distribution of local reaction zone speed, $S_{\mathrm{RZ}}$ , presented as a fraction, $f(S_{\mathrm{RZ}})$ , of all local measurements at each recorded frame. 50^*th*^, 75^*th*^, and 90^*th*^ percentiles of $S_{\mathrm{RZ}}$ shown ( $S_{\mathrm{RZ},50}$ , $S_{\mathrm{RZ},75}$ , $S_{\mathrm{RZ},90}$ ). Representative single-cycle OH*-CL images (image phasing indicated by dotted line) with red vectors indicating the measured displacement of reaction zone boundaries used to calculate $S_{\mathrm{RZ}}$ .

In Figure 15, $S_{\mathrm{RZ}}$ is compared for RIT=−33° , −53° , −95° , −153° . For $\mathrm{RIT}\leq \mathrm{RI}T_{\mathrm{insens}.}$ , a common pattern in the distribution of $S_{\mathrm{RZ}}$ is observed: high initial $S_{\mathrm{RZ}}$ during pilot auto-ignition, followed by relatively low $S_{\mathrm{RZ}}$ during flame propagation. This behavior and the magnitude of $S_{\mathrm{RZ}}$ is similar to previous measurements of port-injected dual-fuel combustion with similar ϕ in the same facility.^40^ For RIT=−33° , the peak $S_{\mathrm{RZ}}$ is retarded and less prominent than for $\mathrm{RIT}\leq \mathrm{RI}T_{\mathrm{insens}.}$ . This is a consequence of the high CCV of combustion phasing for RIT=−33° preventing the pilot ignition of individual cycles from aligning temporally.

Despite a significant decrease in peak AHRR for RIT=−153° relative to RIT=−95°,−53° , $S_{\mathrm{RZ}}$ appears very similar for these conditions. This discrepancy may be related to non-simultaneous pilot ignition and main combustion processes for RIT=−153° (i.e. earlier pilot ignition on right side of combustion chamber, see Appendix D).

## Characterization of the spectrum of premixed NG combustion

**Figure 16.**
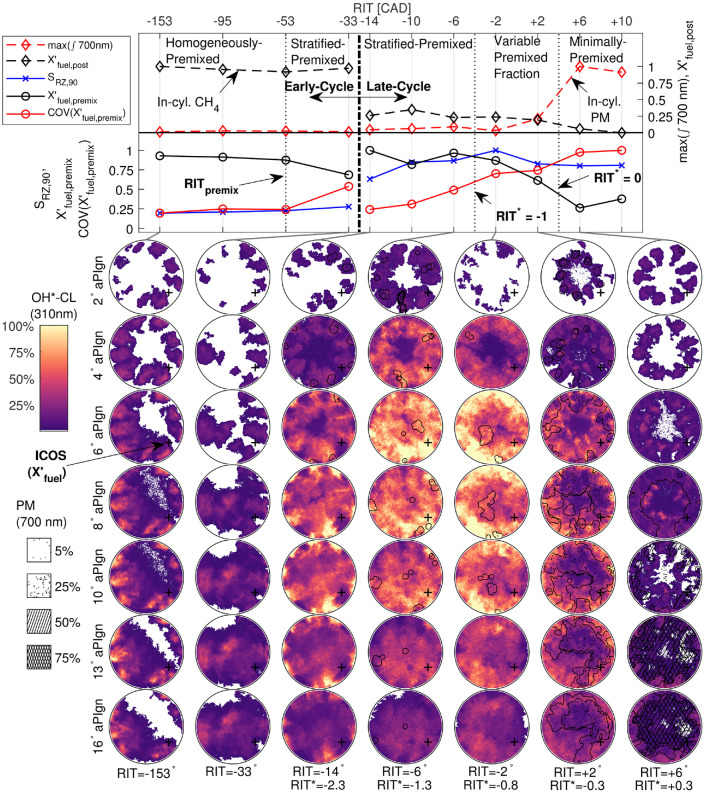
Overview of the spectrum of PIDING combustion with varying NG premixing. Normalized $X\prime_{\mathrm{fuel},\mathrm{premix}}$ and COV $\left(X\prime_{\mathrm{fuel},\mathrm{premix}}\right)$ shown to characterize NG premixing. Normalized $S_{\mathrm{RZ},90}$ shown to characterize fuel conversion rates. Normalized $X\prime_{\mathrm{fuel},\mathrm{post}}$ and ∫700 nm shown to characterize incomplete combustion of CH_4_ and in-cylinder PM, respectively. Representative single-cycle OH*-CL and 700 nm images shown for each regime of PIDING combustion ( RIT=−153°,−33°,−14°,−6°,−2°,+2°,+6° ).

In this section, a summary of combustion behavior for all regimes of PIDING combustion is presented to characterize the spectrum of stratified PIDING combustion. In Figure 16, metrics characterizing NG mixture development, fuel conversion rate, and in-cylinder emissions are presented with representative single-cycle images of OH*-CL (310 nm) and PM (700 nm). NG mixture development is characterized by $X\prime_{\mathrm{fuel},\mathrm{premix}}$ , calculated as the magnitude of $X\prime_{\mathrm{fuel}}$ evaluated at the start of premixed NG combustion ( $\theta_{\mathrm{SOC},\mathrm{NG}}$ ). Fuel conversion rate is characterized by the reaction zone growth rate, $S_{\mathrm{RZ},90}$ . The maximum integrated PM signal from 700 nm imaging (max( ∫700 nm)) and $X\prime_{\mathrm{fuel}}$ measured after combustion ( $X\prime_{\mathrm{fuel},\mathrm{post}}$ ) are used to characterize in-cylinder PM and unburned CH_4_, respectively.

For minimally-premixed combustion, $X\prime_{\mathrm{fuel},\mathrm{premix}}>$ 0 indicates that some NG penetrates past the ignition zones and premixes prior to the start of premixed NG combustion ( $\theta_{\mathrm{SOC},\mathrm{NG}}$ ). The NG injection occurs simultaneously to initiation of premixed NG combustion, so the premixed NG concentration has high cyclic variability (high COV( $X\prime_{\mathrm{fuel},\mathrm{premix}})$ ). NG combustion initiates in the NG jet and subsequently spreads to the premixed NG near the bowl wall.^12,31^ Unburned CH_4_ indicated by $X\prime_{\mathrm{fuel},\mathrm{post}}$ is low because NG premixing is limited. High ∫700 nm and 700 nm imaging demonstrates relatively high PM near the bowl wall is generated subsequent to non-premixed combustion, which agrees with previous pyrometric imaging of similar PIDING combustion conditions.^32^

When $\mathrm{RI}T^{*}$ is reduced from minimally-premixed conditions past $\mathrm{RI}T^{*}=0$ to the variable premixed fraction regime, a greater mass of NG premixes (increasing $X\prime_{\mathrm{fuel},\mathrm{premix}}$ ) prior to $\theta_{\mathrm{SOC},\mathrm{NG}}$ (Figure 8) and there is a significant reduction in COV( $X\prime_{\mathrm{fuel},\mathrm{premix}})$ . This is accompanied by a moderate increase of $X\prime_{\mathrm{fuel},\mathrm{post}}$ , which qualitatively matches an increase of exhaust CH_4_ emissions and an order of magnitude drop in in-cylinder PM ( ∫700 nm).^24^ The transition to the variable premixed fraction regime is also marked by a significant increase in OH*-CL intensity near the piston bowl wall, however $S_{\mathrm{RZ}}$ remains relatively unaffected, likely because the reaction zone growth rate is dominated by injection generated turbulence for a given ϕ and charge temperature (Figure 10).

For RIT previously shown to cause quenching of the pilot by the NG jets ( −6°<RIT≤+2° ), premixed NG combustion initiates over a greater volume close to the bowl wall, which is unique among all investigated operating conditions. Conditions with pilot quenching also feature much higher $S_{\mathrm{RZ}}$ and OH*-CL intensity, which indicates that a bulk or multi-zone reaction initiation occurs, rather than OH*-CL being aligned with the pilot fuel jets, as is characteristic of most PIDING conditions. Pilot quenching also likely contributes to the lowest in-cylinder PM ( ∫700 nm) of all late-cycle operating conditions. High $S_{\mathrm{RZ}}$ and distributed reaction zones are considered likely causes for the high thermal efficiency observed for the late-cycle stratified-premixed regime (and $DI^{2}$ combustion).^16,24^

For late-cycle stratified-premixed conditions ( $\mathrm{RI}T^{*}<-1$ ), peak AHRR and $S_{\mathrm{RZ}}$ reduce with increasing NG residence time ( $\tau_{\mathrm{NG}}$ ) as the COV( $X\prime_{\mathrm{fuel},\mathrm{premix}})$ reduces to a similar level measured for homogeneously premixed conditions despite much shorter NG residence time. For $\mathrm{RI}T^{*}<-1.3$ ( RIT<−6° ), NG injection is too advanced to quench the pilot combustion, so distinct pilot reaction zones are observed in the OH*-CL.

For all operating conditions, COV( $X\prime_{\mathrm{fuel},\mathrm{premix}})$ decreases with increasing $\tau_{\mathrm{NG}}$ , except for RIT=−33° . This unique NG mixture development behavior for RIT=−33° is considered a consequence of NG jet impingement near the piston bowl edge and possibly the influence of squish flow from piston motion. This variability results in weak reaction zones (i.e. low OH*-CL intensity), with irregular structures (high cyclic variability).

A step-change in the behavior of all considered NG mixing and combustion metrics occurs across the transition from late-cycle to early-cycle combustion regimes, where NG jet impingement transitions from inside the piston bowl (late-cycle) to outside the piston bowl (early-cycle). Across this transition, the max(AHRR) and $S_{\mathrm{RZ},90}$ decrease, and $X\prime_{\mathrm{fuel},\mathrm{post}}$ increases significantly, which qualitatively matches the observed increase of exhaust CH_4_ emissions for early-cycle PIDING relative to late-cycle.^24^ Premixed NG near the bowl wall is consumed much later for early-cycle conditions relative to late-cycle conditions due to slower reaction zone propagation (i.e. lower $S_{\mathrm{RZ}}$ ). Early-cycle PIDING combustion is similar to port-injected dual-fuel combustion, except that flame speed (and therefore efficiency) is a function of the RIT , with greater flame speeds achieved with later $\mathrm{SO}I_{\mathrm{NG}}$ .

## Conclusions

In-cylinder imaging of OH*-CL (310 nm) and PM (700 nm) was performed for 11 PIDING operating conditions, representing 5 regimes of stratified PIDING combustion previously identified based on AHRR and emissions behavior.^24^ To support in-cylinder imaging, local measurement of relative molar fuel concentration was performed for reacting and non-reacting engine operation to characterize gaseous fuel mixing evolution. The objectives of this investigation were to: (i) support and refine the previously identified regimes of PIDING combustion and critical injection phasings and (ii) describe the in-cylinder mixing process of direct injected gaseous fuel and its impacts on combustion and in-cylinder pollutant formation processes. Detailed descriptions of the in-cylinder processes of each regime are given in § 4.

The in-cylinder imaging and relative fuel concentration measurements provided an improved understanding all PIDING combustion regimes and critical injection phasings ( $\mathrm{RI}T_{\mathrm{premix}}$ , $\mathrm{SO}I_{\mathrm{NG},\mathrm{trans}}$ , $\mathrm{RI}T^{*}=-1$ , and $\mathrm{RI}T^{*}=0$ ):

1.
$\mathrm{RI}T_{\mathrm{premix}}$ : Measurement of premixed NG concentration at the bowl wall in non-fired experiments indicates that an approximately homogeneous fuel-air mixture is reached 12 ms after $\mathrm{EO}I_{\mathrm{NG}}$ , which corresponds to the NG residence time of $\mathrm{RI}T_{\mathrm{premix}}$ . Thus, for $\mathrm{RIT}<\mathrm{RI}T_{\mathrm{premix}}$ , the emissions are less sensitive to RIT as the mixture is homogeneous. Further, this validates the previously proposed approach for metal engines, in which the sensitivity of CO and CH_4_ to RIT are used to identify a homogeneous charge.
2.
$\mathrm{SO}I_{\mathrm{NG},\mathrm{trans}}$ : When $\mathrm{SO}I_{\mathrm{NG}}$ is advanced past $\mathrm{SO}I_{\mathrm{NG},\mathrm{trans}}$ , the premixed fuel concentration near the bowl wall undergoes a step decrease in magnitude and a step increase in cyclic variability. Imaging results also show high cyclic variability in the reaction zone structures. This demonstrates that adverse mixing occurring when the NG jets align with the piston bowl corner at $\mathrm{SO}I_{\mathrm{NG}}$ is the cause for the rapid deterioration of combustion and emissions performance observed in metal engine experiments. To improve stratified-premixed PIDING combustion, gaseous fuel injection angle and piston bowl geometry (which define $\mathrm{SO}I_{\mathrm{NG},\mathrm{trans}}$ ) should be designed to advance $\mathrm{SO}I_{\mathrm{NG},\mathrm{trans}}$ as much as possible.
3.
$\mathrm{RI}T^{*}$ : With decreasing RIT from minimally-premixed PIDING operation, the magnitude of the premixed NG concentration near the bowl wall begins to increase at $\mathrm{RI}T^{*}=0$ and reaches a maximum value at approximately $\mathrm{RI}T^{*}=-1$ . This indicates that $\mathrm{RI}T^{*}$ (calculated using metal engine measurements) is an appropriate metric to qualitatively characterize the premixed NG fraction, $f_{\mathrm{premix}}$ . These observations also strengthen $\mathrm{RI}T^{*}=-1$ and $\mathrm{RI}T^{*}=0$ as valid boundaries between the stratified-premixed, variable-premixed fraction, and minimally-premixed PIDING combustion regimes.

Several important features of direct-injected gaseous fuel mixing and the corresponding implications for combustion performance and in-cylinder pollutant formation have been identified:

1. **NG mixture evolution:** Regardless of the operating condition or RIT, NG premixing takes place near the piston bowl wall prior to ignition, including operation where the gaseous fuel is injected after the pilot combustion. The evolution of premixed NG concentration near the bowl wall is sensitive to RIT and does not develop monotonically with increasing NG residence time. For late-cycle operation, when $\mathrm{SO}I_{\mathrm{pilot}}$ is before $\mathrm{EO}I_{\mathrm{NG}}$ , the NG premixing processes near the piston bowl wall are additionally influenced by the pilot injection.
2. **High indicated efficiency combustion:** Very short combustion durations in the variable-premixed and stratified-premixed (late-cycle) PIDING combustion regimes (SPC^13^ and $DI^{2}$ ^16,17^ elsewhere) is likely due to very rapid reaction zone growth rates ( >45 m/s) driven by high injection generated turbulence shortly after $\mathrm{EO}I_{\mathrm{NG}}$ . When pilot and NG injections overlap, the pilot combustion is (partially) quenched. This results in more premixing, more uniformly distributed early reaction zones (possible multi-point ignition), and even higher reaction zone growth and heat release rates. For these operating conditions, the highest intensity combustion occurs near the piston bowl wall, which may mitigate wall quenching and CH_4_ emissions; this is a recommended area of focus for future investigation.
3. **Early-cycle PIDING combustion:** For homogeneously-premixed PIDING combustion, flame propagation speeds decrease from approximately 15 to 10 m/s as $\mathrm{SO}I_{\mathrm{NG}}$ is advanced from −52° to −171° aTDC. This results in increased combustion durations and reduced indicated efficiency observed in metal engine experiments. The role of injection-generated turbulence is recommended as an area to be investigated further for high efficiency fully-premixed direct-injected NG combustion.

This investigation has validated the previously identified regimes of PIDING combustion and has augmented them with description of the in-cylinder NG mixture development and its impacts on combustion and pollutant formation processes. Further investigation of NG mixing, pilot-NG interactions, and the implementation of numerical modeling of stratified-premixed PIDING combustion is needed to further support and extend the conclusions presented in the current work. These results, combined with recommended future areas of research offer significant opportunity for developing higher-efficiency gaseous fuel direct-injection combustion strategies with low pollutant emissions.

## Figures and Tables

**Figure A1.**
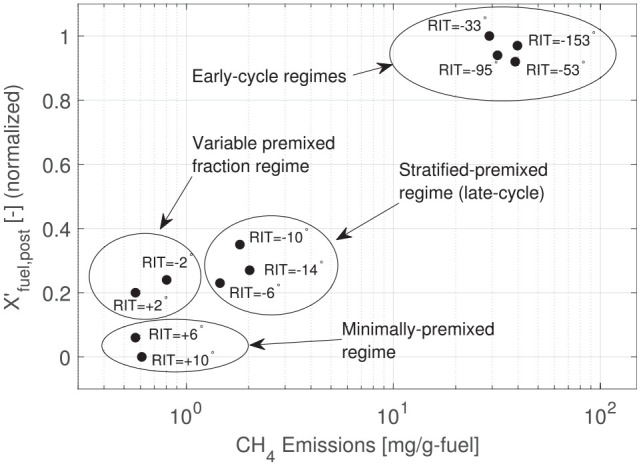
Correlation of post combustion CH_4_ concentration measured in-cylinder ( $X\prime_{\mathrm{fuel},\mathrm{post}}$ , see Appendix C) with exhaust emissions of unburned CH_4_ measured using thermodynamic engine configuration.^24^

**Figure A2.**
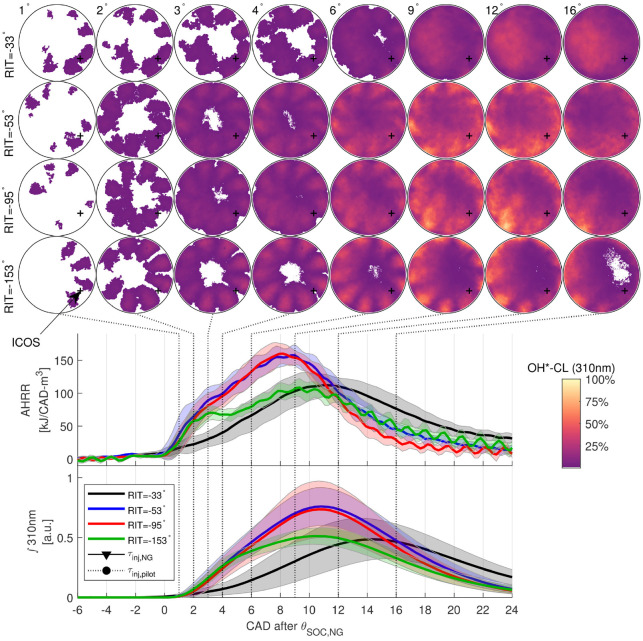
Comparison of ensemble averaged images of OH*-CL (310 nm) with AHRR and ∫310 nm for early-cycle PIDING combustion conditions. Note that all temporal phasing is relative to $\theta_{\mathrm{SOC},\mathrm{NG}}$ .

## Appendix A

### Calculation of X’_fuel_

To characterize the fuel-air mixture development the LaVision Internal Combustion Optical Sensor (ICOS) was implemented. The development and theory of the ICOS is described in detail elsewhere,^38^ and implementation of this instrument in the current experimental facility is described in previous work.^39^ The ICOS measures absorption of light sent via fiber optic cable from a quartz-tungsten-halogen lamp to a 20 mm^3^ measurement volume protruding from the cylinder head. Light introduced to the measurement volume is reflected by a mirrored surface, transmitted back to a second fiber optic cable and 3.4 μ m narrow band-pass filter before reaching the detector. This absorption band measures the C-H vibrational band, characteristic of hydrocarbon fuels, and is related to the fuel molar concentration within the measurement volume using the Beer-Lambert law:

(A.1)
$$N_{\mathrm{fuel}}=-\frac{\ln(\frac{I}{I_{0}})}{L\cdot \sigma }$$

where I is the light intensity measured by the detector, $I_{0}$ is the measured light intensity with no fuel present (measured as the average I from −200° to −180° aTDC for every cycle), σ is the absorption strength coefficient (species-specific), and L is the absorption path length.

To account for broadband IR emission from hot combustion chamber surfaces, an optical chopper modulates the light source at 30 kHz. Thus, the measured intensity, I (equation (A.1)) is the difference between the transmitted intensity with the light on, and the recorded intensity with the light off ( $I=I_{\mathrm{on}}-I_{\mathrm{off}}$ ). The fuel mole fraction, $X_{\mathrm{fuel}}$ , in the ICOS measurement volume is given by:

(A.2)
$$X_{\mathrm{fuel}}(\theta )=\frac{N_{\mathrm{fuel}}(\theta )}{N_{\mathrm{tot},u}(\theta )}$$

where $N_{\mathrm{tot},u}$ is the total molar concentration (including all species) of the unburned mixture in the cylinder, and is assumed to be the same as the mixture within the ICOS measurement volume. $N_{\mathrm{tot},u}$ varies throughout a cycle due to: (i) the changing cylinder volume, $V_{\mathrm{cyl}}$ and (ii) compression of unburned gases by the expanding burned gases following ignition. Assuming ideal gas behavior, $N_{\mathrm{tot},u}$ can be calculated as:

(A.3)
$$N_{\mathrm{tot},u}(\theta )=\frac{\sum n_{i}}{V_{\mathrm{cyl}}(\theta )}=\frac{n_{\mathrm{tot}}}{V_{\mathrm{cyl}}(\theta )}=\frac{P(\theta )}{R\cdot T_{u}(\theta )}$$

where $n_{i}$ and $n_{\mathrm{tot}}$ are the number of mols of the $i^{\mathrm{th}}$ and of all species, respectively. P is the measured cylinder pressure, R is the universal gas constant, and $T_{u}$ is the unburned gas temperature. $T_{u}$ is estimated by assuming isentropic compression of the unburned gases by the cylinder volume change and burned gas expansion, using the relation:

(A.4)
$$T_{u}(\theta )=T_{\mathrm{ref}}\cdot {\left(\frac{P(\theta )}{P_{\mathrm{ref}}}\right)}^{\frac{\gamma -1}{\gamma }}$$

where γ=1.36 , and $P_{\mathrm{ref}}$ and $T_{\mathrm{ref}}$ are evaluated at −170 CAD aTDC and assumed to be the same as the measured inlet manifold conditions at −170 CAD aTDC. When equations (A.1)–(A.4) are combined, the following expression for $X\prime_{\mathrm{fuel}}$ is obtained:

(A.5)
$$X\prime_{\mathrm{fuel}}(\theta )=\left(-\frac{R}{L\cdot \sigma }\right)\cdot \left(\frac{T_{\mathrm{ref}}}{{P_{\mathrm{ref}}}^{\frac{\gamma -1}{\gamma }}}\right)\cdot \left(\frac{\mathrm{In}\left(\frac{I\left(\theta \right)}{I_{o}}\right)}{P{\left(\theta \right)}^{\frac{1}{\gamma }}}\right)$$

The calculation of $N_{\mathrm{tot},u}$ considers the unburned gas temperature, $T_{u}$ (equation (A.4)) and is not valid when the ICOS measurement volume contains the burned gases or reacting mixture (i.e. during and after the oxidation of fuel in the ICOS measurement volume). A sharp drop in $N_{\mathrm{fuel}}$ (i.e. drop in fuel concentration) provides clear indication of the crank angle phasing when the fuel in the ICOS measurement volume is oxidized and therefore the limit of where $X\prime_{\mathrm{fuel}}$ as described above can be applied.

In future work, the spectral, temperature, and pressure sensitivities of σ will be considered to provide quantitative assessment of $X_{\mathrm{fuel}}$ . In the current work however, qualitative comparisons of $X\prime_{\mathrm{fuel}}$ are made so σ can be assumed constant and combined with other parameters into a proportionality constant. This results in the following form of $X\prime_{\mathrm{fuel}}$ :

(A.6)
$$X\prime_{\mathrm{fuel}}(\theta )\alpha \left(\frac{\mathrm{In}\left(\frac{I\left(\theta \right)}{I_{o}}\right)}{P{\left(\theta \right)}^{\frac{1}{\gamma }}}\right)$$

## Appendix B

### Analysis of OH*-Chemiluminescence

Analysis of OH*-CL must consider the mechanisms leading to OH radical production and chemiluminescence as well as the impact of combustion chamber conditions on OH*-CL. The formation of the OH* radical is considered to primarily occur via two reactions ^41^:

(R1)
$$\mathrm{CH}+O_{2}->OH^{*}+\mathrm{CO}$$

(R2)
$$H+O+M->OH^{*}+M$$

The OH*-CL intensity is sensitive to pressure, ϕ , and temperature for CH_4_ flames at relevant cylinder conditions (increasing OH*-CL intensity with decreasing pressure, increasing ϕ , or increasing T) and is commonly used as a measure of local heat release in premixed flames (i.e. R1).^31,41^ OH*-CL is also emitted from the burned gas regions due to the recombination of H and O atoms at elevated temperatures, particularly for lean combustion where there is a higher concentration of O in the combustion products (R2).^41^ The presence of PM has been shown to significantly attenuate OH*-CL, which must be considered when interpreting OH*-CL for non-premixed combustion systems.^32^

## Appendix C

### Comparison of CH_4_ Emissions and X’_fuel, post_

To characterize unburned CH_4_ due to incomplete combustion, local measurement of $X\prime_{\mathrm{fuel}}$ following combustion, $X\prime_{\mathrm{fuel},\mathrm{post}}$ , is compared to exhaust emissions of CH_4_ in Figure A1. The calculation of $X\prime_{\mathrm{fuel}}$ presented in this work (equations (A.1)–(A.5)) used the properties of the unburned gases to estimate the total molar concentration in the cylinder, $N_{\mathrm{tot},u}$ (equation (A.3)). To calculate $X\prime_{\mathrm{fuel},\mathrm{post}}$ , the burned gas properties are assumed to be representative of the entire charge (i.e. a single thermodynamic zone) and are used to estimate $N_{\mathrm{tot},b}$ :

(A.7)
$$N_{\mathrm{tot},b}=\frac{n_{\mathrm{tot}}}{V(\theta )}=\frac{m_{\mathrm{cyl}}}{{\tilde{M}}_{\mathrm{ex}}\cdot V(\theta )}=\frac{\left(m_{\mathrm{air}}+m_{\mathrm{NG}}+m_{\mathrm{diesel}}\right)}{{\tilde{M}}_{\mathrm{ex}}\cdot V(\theta )}$$

where the molar mass, ${\tilde{M}}_{\mathrm{ex}}$ , is estimated assuming complete combustion of the reactants. $X\prime_{\mathrm{fuel}}$ is then calculated as previously described (equation (A.2)), using equation (A.7) for $N_{\mathrm{tot},b}$ . $X\prime_{\mathrm{fuel},\mathrm{post}}$ is calculated as the average of $X\prime_{\mathrm{fuel}}$ over the interval of θ=[30,90] CAD aTDC. Exhaust emissions were measured in a previous investigation of the same operating conditions using the thermodynamic configuration of the engine facility applied in the current work.^24^ Note that due to the significant increase in CH_4_ emissions for early-cycle regimes relative to late-cycle regimes, a log scale is used for the CH_4_ emissions axis to improve legibility.

## Appendix D

### Early-cycle combustion imaging

In Figure A2 ensemble averaged images of OH*-CL are compared for all early-cycle combustion modes (note PM and ∫700 nm not shown due to low PM signal for early-cycle operating conditions). The reaction zone structure and AHRR for $\mathrm{RIT}>\mathrm{RI}T_{\mathrm{insens}.}$ is unique compared to other conditions shown with $\mathrm{RIT}\leq \mathrm{RI}T_{\mathrm{insens}.}$ . Following pilot ignition, OH*-CL images for RIT=−33° in Figure A2 ( θ≥6° after $\theta_{\mathrm{SOC},\mathrm{NG}}$ ) show the pilot reaction zones merging in the center of the combustion chamber. Despite high CCV of RIT=−33° , single-cycle images indicate similar reaction zone structures to the ensemble average (e.g. see Figure 15).

For $\mathrm{RIT}\leq \mathrm{RI}T_{\mathrm{insens}.}$ , a long delay between ignition (indicated by AHRR) and premixed NG combustion near the bowl wall (measured by the ICOS) demonstrates a relatively slow flame propagation process. The AHRR and ∫310 nm indicate very similar combustion for RIT=−53° , −95° , but the magnitude of both decrease significantly for RIT=−153° where systematic asymmetries in the combustion process become prominent.

For RIT=−153° , pilot auto-ignition starts earlier for reaction zones on the right side of the chamber (see 1°−3° after $\theta_{\mathrm{SOC},\mathrm{NG}}$ in Figure A2), followed by higher OH*-CL intensity for reaction zones on the left side of the combustion chamber during main combustion. While the combustion chamber itself is asymmetric (e.g. intake and exhaust valves on the left side of the combustion chamber, see Figure 5), the reason ignition and main combustion are more sensitive to these asymmetries for RIT=−153° remains an open question.

### Notation

**Table:**

Term	Description
γ	Ratio of specific heats
$\eta_{i,g}$	Indicated gross thermal efficiency
θ	Crank angle
$\theta_{\mathrm{SOC},\mathrm{NG}}$	Crank angle phasing of the start of NG combustion
$\theta_{\mathrm{SOC},\mathrm{ICOS}}$	Crank angle phasing when the ICOS detects fuel consumption
σ	Radiative absorption strength coefficient
$\tau_{\mathrm{NG}}$	NG premixing time (in cylinder prior to combustion)
$\tau_{\mathrm{inj},\mathrm{NG}}$	NG injection duration
ϕ	Equivalence ratio
AHRR	Apparent heat release rate
aTDC	After top dead center
$C_{\mathrm{fuel}}$	Local molar fuel concentration
$CA_{50}$	Phasing of 50% indicate heat release
CAD	Crank angle degree
CCV	Cycle-to-cycle variability
DI^2^	Co-direct injection
DISI	Direct-injection spark-ignition
DLSR	Dome-loaded self-relieving regulator
ECU	Engine control unit
$\mathrm{EO}I_{\mathrm{NG}}$	NG end of injection
$f_{\mathrm{premix}}$	Fraction of NG that premixes prior to combustion
GHG	Greenhouse gas
HPDI	High-pressure direct-injection
I	Light intensity measured by ICOS
ICOS	LaVision in-cylinder optical sensor
IR	Infrared
L	ICOS absorption optical path length
$m_{\mathrm{air}}$	Mass of air in cylinder charge
$m_{\mathrm{cyl}}$	Mass of cylinder charge
$m_{\mathrm{diesel}}$	Mass of diesel in cylinder charge
$m_{\mathrm{NG}}$	Mass of NG in cylinder charge
${\tilde{M}}_{\mathrm{ex}}$	Molar mass of burned gases
$N_{\mathrm{fuel}}$	Molar concentration of fuel in cylinder
$N_{\mathrm{tot},b}$	Molar concentration of cylinder burned gases
$N_{\mathrm{tot},u}$	Molar concentration of cylinder unburned gases
NG	Natural gas
OH*-CL	OH*-chemiluminescence
$P_{\mathrm{cyl}}$	Cylinder pressure
$P_{\mathrm{peg}}$	Pegging pressure at intake manifold
PIDING	Pilot-ignited direct-injection natural gas
PM	Particulate matter
R	Universal gas constant
RCEM	Rapid compression/expansion machine
RIT	Relative injection timing
$\mathrm{RI}T^{*}$	Scaled RIT
$\mathrm{RI}T_{\mathrm{crit}}$	RIT distinguishing minimally-premixed PIDING combustion
$\mathrm{RI}T_{\mathrm{insens}.}$	Largest RIT at which emissions show strong sensitivity to RIT
RoPR	Rate of pressure rise
$\mathrm{SO}I_{\mathrm{NG}}$	NG start of injection
$\mathrm{SO}I_{\mathrm{NG},\mathrm{trans}}$	$NG_{\mathrm{SOI}}$ where NG orifice aligns with piston bowl corner
$\mathrm{SO}I_{\mathrm{pilot}}$	Pilot start of injection
SPC	Slightly premixed combustion
$T_{\mathrm{peg}}$	Pegging temperature at intake manifold
TDC	Top dead center
TKE	Turbulent kinetic energy
uHC	Unburned hydrocarbon
V	Cylinder volume
WFS	Westport fuel systems
$X\prime_{\mathrm{fuel}}$	Relative fuel molar concentration (fired operation)
$X\prime_{\mathrm{fuel},\mathrm{NR}}$	Relative fuel molar concentration (non-reactingoperation)
$X\prime_{\mathrm{fuel},\mathrm{post}}$	Average $X\prime_{\mathrm{fuel}}$ measured over θ=[+30°,+90°] aTDC
$X\prime_{\mathrm{fuel},\mathrm{premix}}$	$X\prime_{\mathrm{fuel}}$ measured at $\theta_{\mathrm{SOC},\mathrm{NG}}$
